# Metabolic Dynamics in Skeletal Muscle during Acute Reduction in Blood Flow and Oxygen Supply to Mitochondria: In-Silico Studies Using a Multi-Scale, Top-Down Integrated Model

**DOI:** 10.1371/journal.pone.0003168

**Published:** 2008-09-09

**Authors:** Ranjan K. Dash, Yanjun Li, Jaeyeon Kim, Daniel A. Beard, Gerald M. Saidel, Marco E. Cabrera

**Affiliations:** 1 Center for Modeling Integrated Metabolic Systems, Case Western Reserve University, Cleveland, Ohio, United States of America; 2 Department of Biomedical Engineering, Case Western Reserve University, Cleveland, Ohio, United States of America; 3 Department of Physiology and Biophysics, Case Western Reserve University, Cleveland, Ohio, United States of America; 4 Department of Pediatrics, Case Western Reserve University, Cleveland, Ohio, United States of America; 5 Biotechnology and Bioengineering Center, Medical College of Wisconsin, Milwaukee, Wisconsin, United States of America; 6 Department of Physiology, Medical College of Wisconsin, Milwaukee, Wisconsin, United States of America; IBM Thomas J. Watson Research Center, United States of America

## Abstract

Control mechanisms of cellular metabolism and energetics in skeletal muscle that may become evident in response to physiological stresses such as reduction in blood flow and oxygen supply to mitochondria can be quantitatively understood using a multi-scale computational model. The analysis of dynamic responses from such a model can provide insights into mechanisms of metabolic regulation that may not be evident from experimental studies. For the purpose, a physiologically-based, multi-scale computational model of skeletal muscle cellular metabolism and energetics was developed to describe dynamic responses of key chemical species and reaction fluxes to muscle ischemia. The model, which incorporates key transport and metabolic processes and subcellular compartmentalization, is based on dynamic mass balances of 30 chemical species in both capillary blood and tissue cells (cytosol and mitochondria) domains. The reaction fluxes in cytosol and mitochondria are expressed in terms of a general phenomenological Michaelis-Menten equation involving the compartmentalized energy controller ratios ATP/ADP and NADH/NAD^+^. The unknown transport and reaction parameters in the model are estimated simultaneously by minimizing the differences between available *in vivo* experimental data on muscle ischemia and corresponding model outputs in coupled with the resting linear flux balance constraints using a robust, nonlinear, constrained-based, reduced gradient optimization algorithm. With the optimal parameter values, the model is able to simulate dynamic responses to reduced blood flow and oxygen supply to mitochondria associated with muscle ischemia of several key metabolite concentrations and metabolic fluxes in the subcellular cytosolic and mitochondrial compartments, some that can be measured and others that can not be measured with the current experimental techniques. The model can be applied to test complex hypotheses involving dynamic regulation of cellular metabolism and energetics in skeletal muscle during physiological stresses such as ischemia, hypoxia, and exercise.

## Introduction

Skeletal muscle plays a major role in the regulation of whole-body substrates and energy metabolism, especially under changing physiological conditions such as ischemia (reduced blood flow), hypoxia (reduced oxygen supply), and exercise (increased energy demand). Current experimental techniques provide relatively little *in vivo* data on dynamic responses of muscle metabolite concentrations and metabolic fluxes to such physiological stimuli, especially in subcellular domains, such as mitochondria. To quantitatively analyze available *in vivo* experimental data and predict nonmeasurable dynamic responses, we developed a physiologically-based, multi-scale computational model of skeletal muscle cellular metabolism and energetics. The model is developed here from our previous model of cellular metabolism and energetics in skeletal muscle [1] and incorporates inter-domain transport processes and compartmentalized metabolic reactions of many key chemical species in both cytosol and mitochondria.

Developing a mechanistic computational model of substrates and energy metabolism in complex, multi-scale metabolic systems, such as skeletal muscle, using a detailed, bottom-up systems approach with sparse *in vivo* experimental data—with an objective of achieving a quantitative understanding of the system to physiological perturbations—is a challenging task. Such a modeling approach requires information about the general structural features and catalytic mechanisms of the associated transporters and enzymes, subcellular metabolic pathways and fluxes and their control mechanisms, and tissue/organ specific metabolic characteristics. Such a modeling approach also requires mechanistic models for key functional components of the system (e.g., inter-domain transport processes, glycolysis, TCA cycle, oxidative phosphorylation, fatty acid β-oxidation) to be first individually developed and validated and then integrated to emulate the systems behavior at the molecular, subcellular, cellular, and tissue/organ levels. To avoid this complex approach and facilitate analysis of available sparse *in vivo* experimental data to understand dynamic responses of the system to physiological stresses, approximations are often made to obtain a simplified model of the system that includes key functional components regulating cellular metabolic processes at the desired level of complexity.

A top-down systems approach is an alternative approach which has been previously applied to determine and integrate a representative set of lumped biochemical reactions *in vivo* metabolic systems that incorporate primary substrates and key intermediate metabolites with coupled metabolic energy controllers ATP-ADP and NADH-NAD^+^ [1]–[8]. This approach is similar to the top-down systems approach in metabolic control analysis proposed by Brand and co-workers [9], [10] and is intended to provide an essential or minimal set of stoichiometrically balanced lumped biochemical reactions participating in ATP synthesis within mitochondria from the metabolism of nutrients (e.g., glucose, fatty acids, amino acids). Even with such simplifications, a large number of phenomenological kinetic parameters are introduced in the governing model equations, which must be estimated from available sparse *in vivo* experimental data. To estimate these unknown parameters, constraint-based, robust nonlinear optimization methods are needed, as established in our previous work [1].

To date, no physiologically-based, whole-organ level model of skeletal muscle cellular metabolism and energetics has been developed that can be applied to analyze available *in vivo* experimental data and predict dynamic metabolic responses to physiological stimuli in subcellular compartments, such as mitochondria. Previous models have incorporated some aspects of glycolysis, TCA cycle, oxidative phosphorylation, and fatty acid β-oxidation [3]–[5], [11]–[21]. None of them, however, include sufficient key substrates/metabolites and/or integrate metabolic pathways/reactions that are essential in the regulation of cellular metabolic processes in skeletal muscle *in vivo* at whole-organ level. While our recently developed models of skeletal muscle cellular metabolism and energetics [1] have incorporated key metabolic pathways and reactions, the intracellular cytosolic and mitochondrial compartments were not distinguished. The energy controller pairs ATP-ADP and NADH-NAD^+^ that modulate several key metabolic reactions in the cytosol and mitochondria have different concentrations in these two subcellular domains [6]–[8]. As a consequence, the model may not accurately predict the dynamics of several metabolite concentrations and metabolic fluxes that are critical in the regulation of fuel (carbohydrate, fat, and lactate) metabolism and cellular respiration during physiological stresses such as ischemia, hypoxia, and exercise.

In this paper, a physiologically-based, whole-organ level model of skeletal muscle cellular metabolism and energetics is developed and applied to study dynamic cellular metabolic responses to reduced blood flow and oxygen supply to mitochondria (muscle ischemia). The model, which is extended from our previous model [1], is based on a multi-scale, top-down systems approach [1]–[8], accounts for subcellular compartmentalization, and includes primary substrates (carbohydrates and fats) and key intermediate metabolites and metabolic reactions specific to skeletal muscle metabolic system. The model equations are based on dynamic mass balances of chemical species in capillary blood and tissue cells (cytosol and mitochondria) domains. The model also distinguishes the free and bound forms of O_2_ and CO_2_ transport in the blood and cells. The inter-domain species transport processes are considered either by passive diffusion or by carrier-mediated (facilitated) transport. The metabolic reaction fluxes in the cytosolic and mitochondrial domains are represented by a general phenomenological Michaelis-Menten equation involving the compartmentalized ATP/ADP and NADH/NAD^+^ energy controller ratios. The phenomenological kinetic parameters of the model are estimated by using our recently developed constraint-based, robust nonlinear optimization approach [1]. In this estimation process, we fit the model output to sparse *in vivo* dynamic data on glycolytic and energetic metabolite concentrations from experiments in humans with muscle ischemia previously published [22]. With the estimated optimal parameter values, the model is able to simulate dynamic responses of key chemical species and reaction fluxes to reduced blood flow and oxygen supply to mitochondria associated with the muscle ischemia.

## Materials and Methods

### Model Development

**Figure 1:**
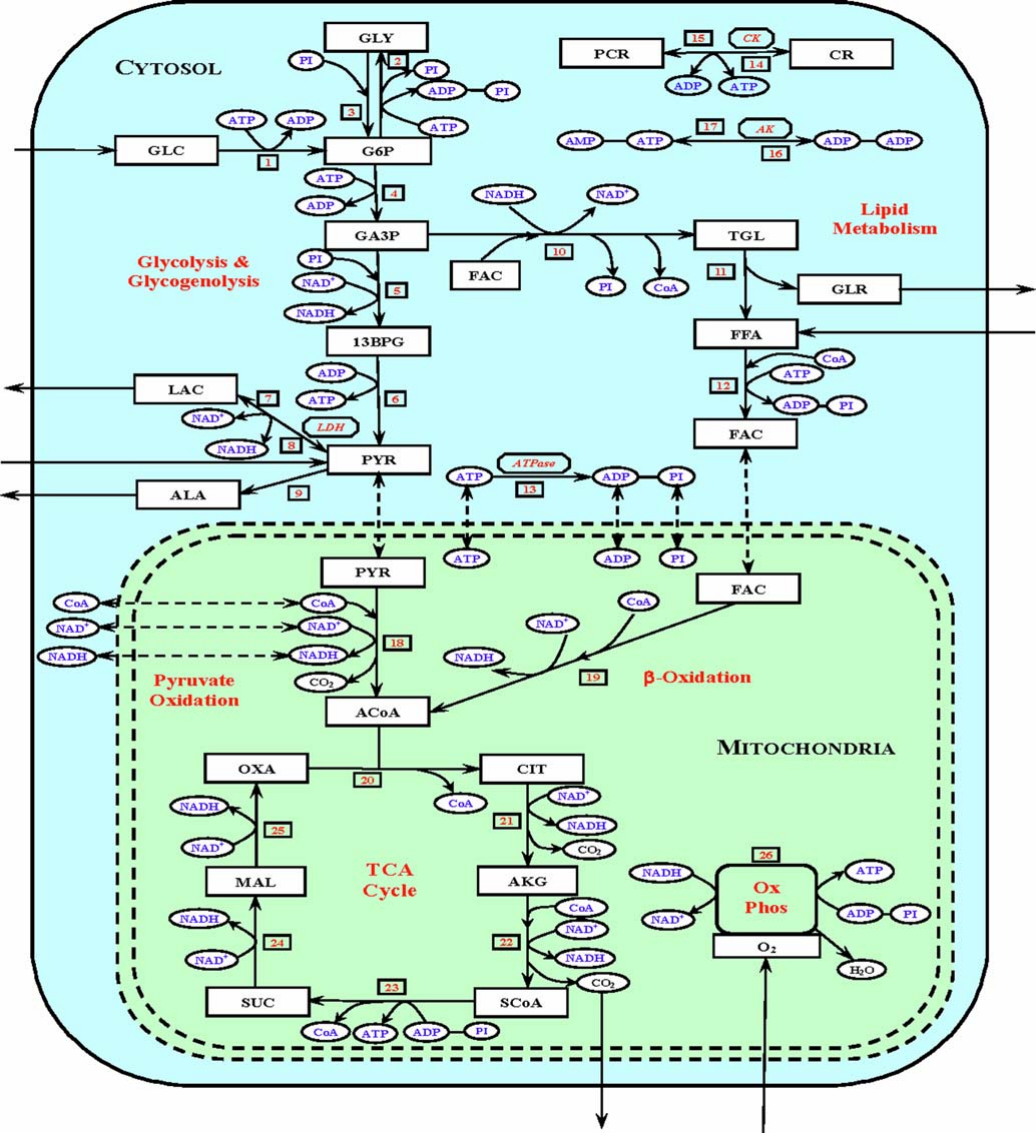
Schematic diagram of biochemical pathways depicting various chemical reactions and species involved in the cellular metabolism of skeletal muscle. The pathways involve 26 lumped reactions among 30 species out of which 8 species (GLC, LAC, PYR, ALA, FFA, GLR, CO_2_ and O_2_) undergo blood-tissue cells exchange. The exchange arrows show the direction of net tissue cells uptake-release rates at normal, resting conditions. The lumped reactions are further compartmentalized into the cytosolic and mitochondrial reactions. These two subcellular domains are assumed to be in rapid equilibrium state (the barrier is shown schematically by double dotted lines), so that the species that are common to both these domains can have the similar dynamics, i.e., *C_mit,j_*(*t*) = *σ_j_.C_cyt,j_*(*t*), where *σ_j_* is the partition coefficient of the species j between cytosol and mitochondria. In this case, the net transport flux of a species j across the cytosol-mitochondria barrier can quickly become negligible at the onset of a physiological perturbation (the transport fluxes are shown by double dotted arrows); 10 species (PYR, FAC, CoA, NADH, NAD^+^, ATP, ADP, PI, CO_2_ and O_2_) exist in both the cytosolic and mitochondrial domains. GLC: glucose, GLY: glycogen, G6P: glucose-6-phosphate, GA3P: glyceraldehyde-3-phosphate, 13BPG: 1,3-biphosphate-glycerate, PYR: pyruvate, LAC: lactate, ALA: alanine, TGL: triglycerides, GLR: glycerol, FFA: free fatty acid, FAC: fatty acyl-CoA, ACoA: acetyl-CoA, CIT: citrate, AKG: α-ketogluterate, SCoA: succinyl-CoA, SUC: succinate, MAL: malate, OXA: oxaloacetate, CoA: coenzyme-A (free), PCR: phosphocreatine, CR: creatine, PI: inorganic phosphate, CO_2_: carbon dioxide, O_2_: oxygen, NADH: reduced nicotinamide adenine dinucleotide, NAD^+^: oxidized nicotinamide adenine dinucleotide, ATP: adenosine triphosphate, ADP: adenosine diphosphate, AMP: adenosine monophosphate.

The first step in our development of a multi-scale, top-down computational model of cellular metabolism and energetics in skeletal muscle was to identify the key intermediate metabolites and regulatory enzymes in the cellular metabolic pathways of skeletal muscle. We then integrated available information on cellular metabolic pathways and fluxes, cellular metabolic control mechanisms, catalytic enzyme kinetic mechanisms, subcellular compartmentation and metabolites volumes of distributions, inter-domain transport mechanisms, and skeletal muscle tissue-specific metabolic characteristics. A simplified map of the compartmentalized cellular metabolic pathways in skeletal muscle is shown in Figure 1. The lumped biochemical reactions in the metabolic pathways are generated by stoichiometrically coupling several sequential elementary reactions. These reactions include the compartmentalized metabolic energy controller pairs ATP-ADP and NADH-NAD^+^ whose ratios are known to modulate (fine tune) the reaction fluxes [2], [23] in the subcellular compartments. Many of these lumped reactions are considered irreversible for which the resting Gibbs free energy (Δ*G*) is high and negative in favor of product formation [24]. As a part of a general formalism for modeling *in vivo* metabolic systems (nonequilibrium open systems), the reversible reactions like lactate dehydrogenase (LDH), creatine kinase (CK), and adenylate kinase (AK) were decomposed into two separate irreversible reactions with distinct kinetics [1].

### Dynamic mass balance equations

**Figure 2:**
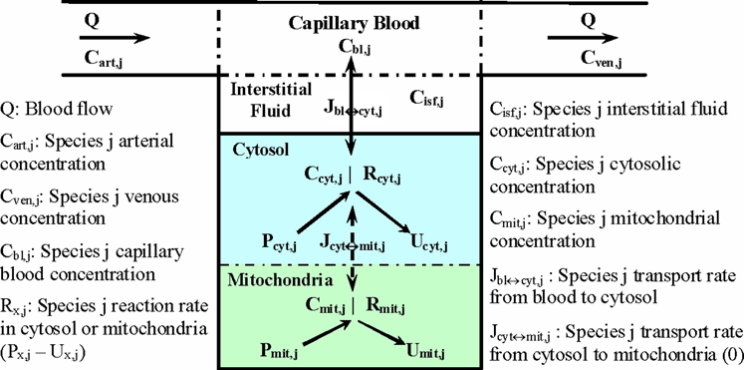
Schematic diagram of the structure of the model for blood-tissue cells exchange and cellular metabolism in skeletal muscle. The compartments are assumed to be perfectly mixed, and the capillary blood and tissue ISF regions are assumed to be in phase-equilibrium with each other, so that *C_isf,j_* = *C_bl,j_* = *C_ven,j_* for any chemical species *j*. The tissue cells domain is further compartmentalized into the cytosolic and mitochondrial domains with the chemical species having the similar dynamics in these two subcellular domains, so that *C_mit,j_*(*t*) = *σ_j_C_cyt,j_*(*t*), where *σ_j_* is the partition coefficient of the species j between cytosol and mitochondria. The model accounts for 30 chemical species in the tissue cells. A total of 8 species (GLC, LAC, PYR, ALA, FFA, GLR, CO_2_ and O_2_) undergo blood-tissue cells exchange; 10 species (PYR, FAC, CoA, NADH, NAD^+^, ATP, ADP, PI, CO_2_ and O_2_) exist in both the cytosolic and mitochondrial domains with a negligible transport flux (*J_cyt↔mit,j_*≈0). For details, see the caption of Figure 1.

The dynamic mass balance equations are based on a multi-domain model structure for skeletal muscle consisting of a spatially-lumped capillary blood domain which exchanges nutrients and metabolic waste products with a spatially-lumped domain of tissue cells (Figure 2). Although these two domains are separated by the interstitial fluid (ISF) space, we assume phase-equilibrium of chemical species between the blood and ISF domains, and consider them together as the “blood” domain. Furthermore, the tissue cells domain is compartmentalized into the cytosolic and mitochondrial domains. The chemical species are assumed to be distributed in these two subcellular domains as per their mass fractions and volumes of distributions (Table 1). Here, the mass fraction of a species in a particular domain is defined as the fractional amount of the species in that domain in comparison to the total amount of the species in the whole muscle tissue cells. The volume of distribution of a species in a particular domain is defined as the anatomical volume of the domain plus the binding space of the domain for the species. In addition, the species that are common to both of these domains are assumed to have the similar dynamics in these two domains, because the species transport processes between these two domains can be sufficiently fast [5]. Consequently, a change in the species concentration in one domain will be proportional to the change in the species concentration in the other domain: *C_mit,j_*(t) = *σ_j_.C_cyt,j_*(t), where *σ_j_* is the equilibrium concentration ratio (or partition coefficient) of species j between mitochondria and cytosol. In the present model, a total of 10 chemical species (pyruvate, fatty acyl-CoA, CoA, ATP, ADP, inorganic phosphate, NADH, NAD^+^, O_2_, and CO_2_) are considered to exist in both the cytosolic and mitochondrial compartments with negligible transport flux between the compartments.

**Table 1:** Average species concentrations in the muscle tissue cells [1] and their compartmentalization (distribution) into cytosol and mitochondria at normal, resting steady-state conditions.*

*Species*	*Tissue Cells (100%)*		*Cytosol (90%)*		*Mitochondria (10%)*	
	*Concentration (mmol/kg cell ww)*	*References*	*Mass* # *(%)*	*Concentration (mmol/kg cyto ww)*	*Mass* # *(%)*	*Concentration (mmol/kg mito ww)*
GLC	0.5	[22], [33], [38], [39], [46]–[50]	100	0.5556	0	0
GLY	95.0	[33], [38], [46], [47], [49], [51]–[53]	100	105.56	0	0
G6P	0.25	[22], [33], [46]–[50]	100	0.2778	0	0
GA3P	0.08	[33], [46], [48]	100	0.08889	0	0
13BPG	0.08	[33], [46], [48]	100	0.08889	0	0
PYR	0.05	[22], [38], [39], [46]–[48], [50]	95	0.0528	5	0.025
LAC	0.78	[22], [33], [38], [39], [46], [48], [50]	100	0.8667	0	0
ALA	1.3	[38], [53]–[55]	100	1.4444	0	0
TGL	15.0	[48], [51], [52]	100	16.667	0	0
GLR	0.065	[33], [47], [49], [52], [56]	100	0.07222	0	0
FFA	0.45	[33], [47], [49], [52], [56]	100	0.50	0	0
FAC	0.0035	[49], [57]	95	0.003694	5	0.00175
ACoA	0.002	[33], [47], [55]	0	0	100	0.02
CIT	0.095	[38], [39], [48], [53]	0	0	100	0.95
AKG	0.0125	[38], [39]	0	0	100	0.125
SCoA	0.125	[38], [39], [48], [53]	0	0	100	1.25
SUC	0.095	[38], [39], [48], [53]	0	0	100	0.95
MAL	0.095	[38], [39], [48], [53]	0	0	100	0.95
OXA	0.003	[38], [39], [48], [53]	0	0	100	0.03
CoA	0.02	[33], [47], [55]	80	0.01778	20	0.04
CO2 (F)	1.40 (46 mmHg)	[26]	90	1.40 (46 mmHg)	10	1.40 (46 mmHg)
CO2 (T)	15.427	[26]	90	15.427	10	15.427
O2 (F)	0.0338 (25 mmHg)	[26]	90	0.0338 (25 mmHg)	10	0.0338 (25 mmHg)
O2 (T)	0.49	[26]	90	0.49	10	0.49
PCR	21.0	[33], [38], [42], [46], [48], [50], [53], [58]	100	23.333	0	0
CR	10.5	[33], [38], [42], [46], [48], [50], [53], [58]	100	11.667	0	0
PI	2.75	[42], [46], [48]	90	2.75	10	2.75
NADH	0.05	[46], [48], [50], [53]	0.5	0.2778E-3	99.5	0.4975
NAD^+^	0.45	[46], [48], [50], [53]	30	0.15	70	3.15
ATP (T)	6.2	[22], [33], [46]–[48], [50], [53], [58]	86	5.924	14	8.68
ADP (T)	0.8	[22], [46], [48], [50], [53], [58]	2	0.01778	98	7.84
AMP (T)	0.04	[22], [46], [48], [50], [53], [58]	100	0.04444	0	0

* The species concentrations (mM or mmol/kg tissue cells ww) are converted from the experimental data (mmol/kg tissue cells dw) in the literature by multiplying a factor 0.25 kg tissue cells dw/kg tissue cells ww [33]; for unit density, kg ww = L and mmol/kg ww = mmol/L = mM. The cytosolic and mitochondrial species concentrations are calculated from the species concentrations in the muscle tissue cells according to their approximate volumes of distributions and mass fractions; species nomenclature is adopted from Ref. [1] (also see the caption to Figure 1); dw denotes dry weight, ww denotes wet weight.

# The mass fractions are set to have reasonable compartmentalized species concentrations and mass action ratios consistent with available information from the literature: C_cyt,LAC_/C_cyt,PYR_ = 16.4, C_cyt,NAD+_/C_cyt,NADH_ = 540, C_mit,NAD+_/C_mit,NADH_ = 6.3, C_cyt,PCR_/C_cyt,CR_ = 2, C_cyt,ATP_/C_cyt,ADP_ = 333.2, C_mit,ATP_/C_mit,ADP_ = 1.11, *K*_LDH_ = (C_cyt,LAC_/C_cyt,PYR_)* (C_cyt,NAD+_/C_cyt,NADH_) = 8856, *K*_CK_ = (C_cyt,CR_/C_cyt,PCR_)*(C_cyt,ATP_/C_cyt,ADP_) = 166.6, and *K*_AK_ = (C_cyt,ADP_)^2^/(C_cyt,ATP_C_cyt,AMP_) = 1.2E-3. The cytosolic and mitochondrial PI concentrations are set at equal value with the assumption of a negligible pH gradient across the mitochondrial membrane.

The dynamic mass balance of a chemical species *j* in the spatially-lumped blood domain has the following general form:
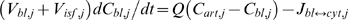
(1)where *C_art,j_* is the arterial species concentration; *C_bl,j_* is the capillary blood species concentration (equal to the venous species concentration *C_ven,j_*); *V_bl,j_* and *V_isf,j_* are the volumes of distribution of species *j* in blood and ISF, and *Q* is the regional blood flow; *J_bl↔cyt,j_* is the net transport flux (mass per unit time) across the blood-cytosol exchange barrier (consisting of capillary membrane, ISF, and tissue cell membrane).

The dynamic mass balance of the chemical species j in the spatially-lumped tissue cells domain (cytosol and/or mitochondria) has the following general forms:
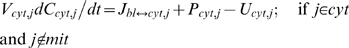
(2a)

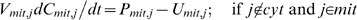
(2b)

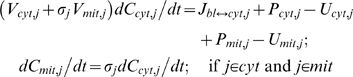
(2c)where
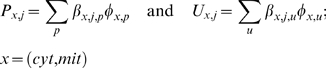
(2d)


Here *C_x,j_* is the species concentration in domain *x* (cytosol/mitochondria); *V_x,j_* is the volume of distribution of species *j* in domain *x*; *P_x,j_* and *U_x,j_* are the production and utilization of species *j* in domain *x*; *φ_x,p_* and *φ_x,u_* are the reaction fluxes of the reactions processes that produce and utilize species *j* in domain *x*; *β_x,j,p_* and *β_x,j,u_* are the corresponding stoichiometric coefficients. For chemical species which are in the tissue cells (cytosol and/or mitochondria) but not in the capillary blood, the transport flux *J_bl↔cyt,j_* is zero.

The dynamic mass balance equations for all the chemical species in blood, cytosol and mitochondrial domains can be rewritten from our previous model of skeletal muscle metabolism [1] by accounting for the species compartmentalized volumes of distributions as laid out in Eqs. (1) and (2a–2c). The dynamic mass balance equations for O_2_ and CO_2_ in these domains are developed by considering their distinct transport and binding mechanisms [25], [26]. The detailed mass balance equations of the chemical species, including O_2_ and CO_2_, are provided in Materials S1.

### Transport and reaction flux equations

The reversible transport flux *J_bl↔cyt,j_* (mass per unit time) of the species *j* across the blood-cytosol exchange barrier is related to the concentrations *C_bl,j_* and *C_cyt,j_* by
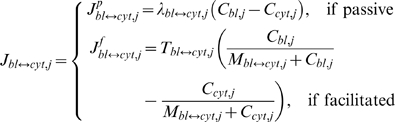
(3)where *λ_bl↔cyt,j_* is the effective permeability surface area product for diffusive mass transport across the barrier (for passive transport); *T_bl↔cyt,j_* is the maximal transport flux across the barrier (*T*_max_) and *M_bl↔cyt,j_* is the corresponding Michaelis-Menten (M-M) constant (*M_m_*) (for facilitated transport). The flux expressions (3) satisfy the thermodynamic equilibrium conditions across the barrier.

The species involved in the blood-cytosol exchange are glucose, lactate, pyruvate, alanine, glycerol, free fatty acid, CO_2_, and O_2_ (Table 2). The transport processes may be passive or carrier-mediated (facilitated) (Eq. 3). The transport of glucose, pyruvate/lactate, and free fatty acid across the sarcolemma of skeletal muscle is facilitated via the GLUT4 [27], MCT1 or MCT4 [28], [29], and FABP or FAT/CD36 [30] proteins, respectively. The sarcolemmal transport of the remaining species (alanine, glycerol, CO_2_, and O_2_) is considered to be passive. For the 10 species that exist in both cytosol and mitochondria, the inter-domain transport flux *J_cyt↔mit,j_* is zero.

**Table 2:** Average muscle tissue cells uptake-release rates (mmol/min) and blood species concentrations (mM) at normal, resting steady-state conditions from the literature.*

*Species*	*Arterial Concentrations, C_art_*	*Venous Concentrations, C_ven_*	*UR rates, Q(C_art_−C_ven_)*	*References*
GLC	5.0	4.7833	0.195	[33], [38], [52], [54], [57], [59]
PYR	0.075	0.0617	0.012	[33], [38], [52], [54], [57], [59]
LAC	0.5	0.6111	−0.10	[33], [38], [52], [54], [57], [59]
ALA	0.25	0.3333	−0.075	[38], [39], [54], [59]
GLR	0.04	0.0483	−0.0075	[33], [49], [52], [56], [57], [59]
FFA	0.7	0.6167	0.075	[33], [49], [52], [56], [57], [59]
CO_2_ (T)	23.405	25.47	−1.86# (−41.66 ml/min)	[38], [52], [54], [57], [59]
CO_2_ (F)	1.22 (40 mmHg)	1.33 (43.55 mmHg)	NA	[25], [26]
O_2_ (T)	9.235	6.556	2.41# (54.0 ml/min)	[38], [52], [54], [57], [59]
O_2_ (F)	0.135 (100 mmHg)	0.0491 (36.37 mmHg)	NA	[25], [26]

# *Physiological constrains*: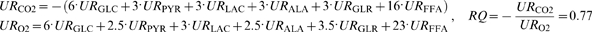.

* A resting blood flow of 0.9 L/min corresponding to the two-leg quadriceps femoris muscle is used to calculate the muscle tissue cells uptake-release (UR) rates from the arterio-venous (AV) differences. The venous species concentrations and uptake-release rates are tuned further to satisfy the physiological constrains^#^ stated above, just below the table. (These well-compiled data are adapted from Table 2, Ref. [1].)

The lumped metabolic reactions of skeletal muscle cellular metabolism and energetics can be considered as special cases of a general, irreversible, multi-reactant multi-product enzymatic reaction coupled with the metabolic energy controller pairs:

**Figure 3:**
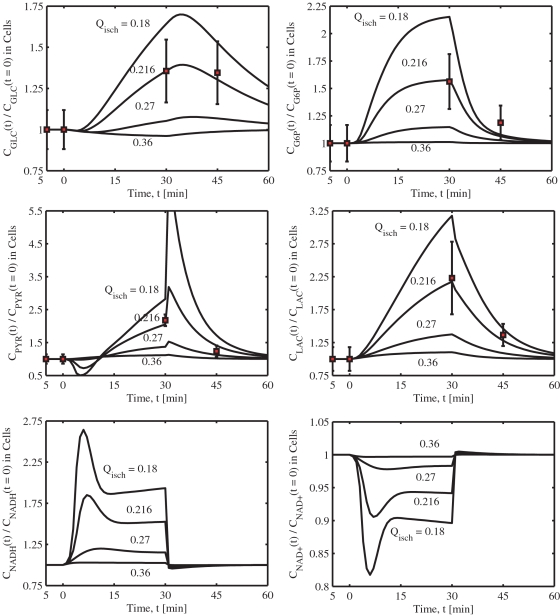
Model-predicted dynamic responses of glycolytic metabolite concentrations and redox states in the muscle tissue cells during the resting, ischemia and recovery periods with varying levels of blood flow reduction and their comparison to the experimental data of Katz [22]. The responses were computed using the estimated optimal parameter values with the ischemia protocol of −5 to 0 min of resting, 0 to 30 min of ischemia, and 30 to 60 min of recovery. The muscle blood flow *Q* is reduced as a step from 0.9 L/min at rest to *Q*_isch_ = 0.36, 0.27, 0.216, 0.18 L/min at the onset of ischemia and returned to 0.9 L/min at the onset of recovery. The lines represent the model simulation results with the symbols representing the experimental data points (mean±SD) corresponding to *Q*_isch_ = 0.216 L/min (∼76% blood flow reduction). The metabolites concentrations are shown in normalized form, normalized with respect to the resting metabolites concentrations. The concentrations in the tissue cells are calculated based on the formula: *C_cl_* = (*V_cyt_C_cyt_*+*V_mit_C_mit_*)/*V_cl_*.

where P_1_ and P_2_ are ATP and ADP or vice-versa (PS±:
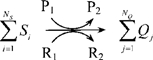
(4) phosphorylation state); R_1_ and R_2_ are NADH and NAD^+^ and vice-versa (RS±: redox state). The corresponding coupled phosphorylation and redox reactions are ATP→ADP (PS+) and/or NADH→NAD^+^ (RS+) or vice-versa (PS- and RS-). Assuming a phenomenological, single-step enzyme kinetic mechanism [31], the flux expression for the lumped metabolic reaction can be written as (see Ref. [1] for detailed description):
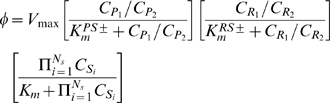
(5)where *V*_max_ and *K_m_* are the phenomenological maximal/limiting velocity and M-M parameter of the reaction; 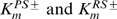 are the phenomenological M-M parameters for the coupled phosphorylation and redox reactions; C_P1_/C_P2_ is the phosphorylation ratio: C_ATP_/C_ADP_ (PS+) or C_ADP_/C_ATP_ (PS-), and C_R1_/C_R2_ is the redox ratio: C_NADH_/C_NAD+_ (RS+) or C_NAD+_/C_NADH_ (RS-). So the reaction fluxes are explicitly regulated by the coupled energy controller ratios C_P1_/C_P2_ and/or C_R1_/C_R2_ (if coupled).

The flux expressions for all the lumped metabolic reactions in the subcellular compartments of cytosol and mitochondria can be rewritten from our previous model of skeletal muscle metabolism [1] in terms of the compartmentalized metabolites concentrations and energy controller ratios ATP/ADP and NADH/NAD^+^. The details are provided in Materials S2.

In summary, in this mathematical model of skeletal muscle cellular metabolism and energetics, the number of chemical species (primary substrates, intermediate metabolites, and energy controllers) in the tissue cells domain (cytosol and mitochondria) is 30, which participate in 26 metabolic reactions. The number of chemical species that exist in both blood and cells is 8, and that in both cytosol and mitochondria is 10. Accordingly, the model includes 8 *J_bl↔cyt,j_* transport fluxes characterized by 4 *λ*, 4 *T*_max_ and 4 *M*_m_ parameters, and 26 *φ* reaction fluxes characterized by 26 *V*_max_, 27 *K*_m_ (1 *K*_m_ for G6P inhibition of the hexokinase reaction; see Reaction 1, Materials S2), 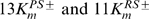 parameters (as in our previous model [1]). Besides, there are 10 partition coefficients *σ* for the chemical species that exists in both cytosol and mitochondria (since the 10 *J_cyt↔mit,j_* transport fluxes are considered negligible). Therefore, the present model is characterized by a total of 99 unknown parameters that need to be estimated to fit the model outputs to the available *in vivo* experimental data. For convenience, the transport and reaction fluxes are written here in vector form: 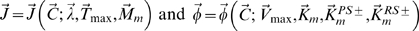, where the parameter vector for these transport and reaction fluxes is denoted by 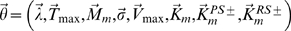; 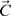 is the concentration vector. Note that the 12 transport parameters (4 *λ*, 4 *T*_max_ and 4 *M*_m_) can be estimated here uniquely from the resting, steady-state transport flux-concentration relationships (Table 3) [1]. The 10 partition coefficients (10 *σ*) can be estimated uniquely from the resting, steady-state species concentration ratios between the mitochondria and cytosol (*σ_j_* = *C_mit,j_*/*C_cyt,j_*) (Table 3).

**Table 3:** Optimal model parameter values for the inter-domain transport fluxes determined from the steady-state parameter estimation process.

I. Blood-cytosol passive or carrier-mediated (facilitated) transport fluxes (mmol/min) and parameters: 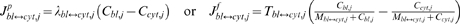				
*Species*	*λ_bl↔cyt_ (ml/min)*	*T_bl↔cyt_ (mmol/min)*	*M_bl↔cyt_ (mmol/L)*	*J_bl↔cyt_ (mmol/min)*
GLC	N/A	0.4106	2.5 [27]	0.195
PYR	N/A	1.5127	1.0 [28], [29]	0.012
LAC	N/A	2.5769	5.0 [28], [29]	−0.10
ALA	0.0675	N/A	N/A	−0.075
GLR	0.3142	N/A	N/A	−0.0075
FFA	N/A	1.5589	1.0	0.075
CO2	24.926	N/A	N/A	−1.86
O2	157.1	N/A	N/A	2.41

II. Cytosol-mitochondrial species partition coefficients (unitless): *σ_cyt↔mit,j_* = *C_mit,j_*/*C_cyt,j_*										
*Species*	PYR	FAC	CoA	PI	ATP	ADP	NADH	NAD^+^	O2	CO2
*σ_cyt↔mit,j_*	0.4737	0.4737	2.25	1.0	1.465	441.0	1791.0	21.0	1.0	1.0

III. Anatomical volumes and muscle blood flow			
*Parameter*	*Definition*	*Value and Unit*	*Reference*
*V_mus_*	Total volume of the active muscle	4.0 kg wet weight (L)	estimated
(*V_bl_*,*V_isf_*, *V_cyt_*, *V_mit_*)	Volumes of blood, ISF, cytosol and mitochondria	(0.07*V_m_*, 0.13*V_m_*, 0.72*V_m_*, 0.08*V_m_*)	calculated
*Q*	Muscle blood flow at rest for two legs	0.9 L/min	[33], [57], [59]
*Q*_isch_	Muscle blood flow during ischemia	0.216 L/min	estimated

The table also includes the anatomical volumes and muscle blood flow at rest (fixed) and during muscle ischemia (estimated).*

* The blood-cytosol transport fluxes satisfies *J_bl↔cyt,j_* = *UR_j_* = *Q(C_art,j_−C_ven,j_)* at resting, steady-state (the UR rates column in Table 2). The subcellular cytosolic and mitochondrial domains are assumed to be in rapid equilibrium state so that the cytosol-mitochondria transport fluxes are considered negligible and the metabolites common to both of these domains are considered to have the similar dynamics (i.e., *C_mit,j_*(*t*) = *σ_j_.C_cyt,j_*(*t*)). Thus the cytosol-mitochondria metabolites partition coefficients *σ_j_* are calculated based on the resting, steady-state metabolite concentrations in these two subcellular domains. The estimates of the transport flux parameters differ from those obtained in our previous work [1] because of the re-estimation of these parameters due to subcellular compartmentalization.

### Model simulation

With specified parameter values, the mathematical model is solved numerically to simulate dynamic responses of the system to ischemia produced by reducing muscle blood flow. Typically, the initial conditions: 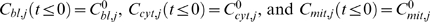 are assumed to be at a normal, resting steady-state. These are fixed based on average resting species concentrations gathered from various literature sources on skeletal muscle cellular metabolism that are consistent with the resting, steady-state flux-concentration relationships (Tables 1 and 2). The species mass fractions and volumes of distributions in the subcellular cytosolic and mitochondrial domains are set to have appropriate phosphorylation and redox potentials in the cytosol and mitochondria. For numerical solution of this stiff initial-value problem, a robust implicit integrator DLSODES (https://computation.llnl.gov/casc/odepack/odepack_home.html; http://www.netlib.org/odepack; [32]) is used. Specifically, the Gear's implicit integration method based on backward difference formula (BDF) is most suitable for this problem. An absolute and relative error of tolerance of 10^−10^ guarantees high accuracy and convergence of the iterative solutions of the ODEs. The DLSODES solver is usually very fast; a typical simulation of this problem using the DLSODES solver in a standard desktop computer (Intel Xeon or Core 2 Duo CPU 5160 @ 3 GHz) takes only about 5 seconds of the CPU time. As a check, the numerical solutions of the initial value problem were also obtained using the ODE15S solver in MATLAB (http://www.mathworks.com) with the similar tolerance levels. These solutions were of comparable accuracy with that obtained using the DLSODES solver.

### Parameter Estimation

The large number of unknown parameters of this model are estimated by comparing model outputs to *in vivo* experimental data of Katz [22] with the optimization procedure described previously [1]. The data consist of key metabolites concentration dynamics measured during circulatory occlusion and recovery (reperfusion) in human skeletal muscle at the whole tissue-organ level [22]. Specifically, the data is based on the biopsy measurements of glucose and glycolytic/glycogenolytic intermediates (i.e., glucose 6-phosphate, pyruvate, and lactate) and creatine and high-energy phosphates (i.e., phosphocreatine, inorganic phosphate, ATP, ADP, and AMP) in quadriceps femoris muscle tissue at rest, after 30 minutes of ischemia, and after 15 minutes of reperfusion. The tissue metabolite contents or concentrations were measured in the units of mmol/kg dry weight. For our analysis, these concentrations are converted to the units of mmol/kg wet weight (mmol/L or mM) by multiplying a conversion factor of 0.25 kg dry weight/kg wet weight, corresponding to the muscle tissue [33]. Consistent with the experimental measurements of Katz [22], the following ischemia protocol is used for model simulations and parameter estimation: the muscle blood flow is reduced from *Q* = 0.9 L/min at rest (t<0 min) to *Q* = *Q*_isch_ (unknown) at the onset of ischemia (0≤t≤30 min), and then increased to the starting level at the onset of recovery (t>30 min). The active muscle volume (*V*_mus_) and ischemic muscle blood flow (*Q*_isch_) responsible for the predicted metabolic dynamics in skeletal muscle during ischemia and recovery were not known from the experimental study of Katz [22]. Therefore, *V*_mus_ and *Q*_isch_ are also included as the unknown parameters for estimation, making the total number of unknown parameters in the model to 101. This is an increase of 10 unknown parameters (10 *σ*) from our previous model [1].

With this relatively sparse data and large number of unknown parameters, the parameter estimation problem is ill-conditioned and under-determined. Nevertheless, an efficient estimation method is devised based on our earlier work [1] to obtain physiologically reasonable parameter values that minimize the sum of squared differences between the available experimental data and corresponding model outputs. The estimation procedure proceeds in two main stages. In the first stage, the published normal, resting species concentration data (Tables 1 and 2) are used to evaluate the resting transport and reaction fluxes from a steady-state flux balance analysis (Tables 2, 3 and 4). From the resting flux-concentration relationships: Eqs. (3) and (5), the preliminary estimates of the transport and reaction parameters are obtained. The preliminary estimates of the 10 partition coefficients are obtained from the resting, steady-state species concentration ratios between mitochondria and cytosol (*σ_j_* = *C_mit,j_*/*C_cyt,j_*) (Table 3). In the second stage, the dynamic species concentration data [22] together with the resting, steady-state flux balance equations as equality constraints are used to obtain the optimal parameter estimates (Tables 3 and 4).

**Table 4:** Resting, steady-state metabolic reaction flux rates and corresponding optimal estimates of the 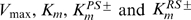 parameters that govern the metabolic reaction flux rates.*

*Metabolic Reactions*	*Flux rates (mmol/min)*	*V_max_ (mmol/min)*	*K_m_ (mmol/L)^n^*	*K^PS±^_m_*(unitless)**	*K^RS±^_m_*(unitless)**
1. Glucose Utilization	0.195	1.1171	6.9E-2, 0.224^##^	334.13 (+)	0
2. Glycogen Synthesis	0.25^#^	0.8006	0.1641	337.69 (+)	0
3. Glycogen Utilization	0.25^#^	50.371	307.178	0.7473 (−)	0
4. G6P Breakdown	0.195	39.615	27.778	337.055 (+)	0
5. GA3P Breakdown	0.3825	43.331	14.354	0	540.233(−)
6. Pyruvate Production	0.3825	959.85	2.1199	0.3002 (−)	0
7. Pyruvate Reduction	25^#^	101.707	0.1575	0	3.904E-05(+)
8. Lactate Oxidation	24.9^#^	3494.12	59.988	0	539.170 (−)
9. Alanine Production	0.075	7.5752	5.2779	0	0
10. TGL Synthesis	0.0075	5.7349	3.214E-2	0	1.25E-2 (+)
11. Lipolysis	0.0075	1.516E-2	17.029	0	0
12. FFA Activation	0.0975	19.705	0.8896	333.08 (+)	0
13. ATP Hydrolysis	15.217	35.961	15.967	333.14 (+)	0
14. PCR Breakdown	100^#^	19822.6	22.091	0.3025 (−)	0
15. PCR Synthesis	100^#^	202.01	0.1142	333.425 (+)	0
16. AMP Utilization	50^#^	254.64	6.872E-02	333.297 (+)	0
17. AMP Production	50^#^	2064.73	1.214E-02	7.063E-2 (−)	0
18. Pyruvate Oxidation	0.2195	0.5043	1.441E-04	0	6.383 (−)
19. FAC Oxidation	0.075	25.995	1.197E-2	0	6.430 (−)
20. Citrate Production	0.8195	83.932	6.085E-2	0	0
21. AKG Production	0.8195	3.7097	1.273	0	5.917 (−)
22. SCoA Production	0.8195	161.242	0.5007	0	5.987 (−)
23. Succinate Production	0.8195	3.1152	2.5616	0.4755 (−)	0
24. Malate Production	0.8195	1.6616	2.024E-02	0	6.238 (−)
25. Oxaloacetate Production	0.8195	1.9415	7.964E-02	0	7.509 (−)
26. Oxygen Utilization	2.41125	258.154	7.696E-04	5.1083 (−)	2.341(+)

* The metabolic reaction flux rates are calculated based on resting, steady-state flux balance analysis (see Table 3, Ref. [1], for details). The flux rates with “#” can not be determined uniquely, and hence are set at values consistent with the other flux values (e.g., set a large value for the fast equilibrium reversible reactions like CK, AK and LDH). For *K^PS±^_m_*and*K^RS±^_m_*parameters, “+” indicates that the energy controller ratio is C_ATP_/C_ADP_ or C_ATP_/C_AMP_ or C_NADH_/C_NAD+_, “−” indicates that the energy controller ratio is C_ADP_/C_ATP_ or C_AMP_/C_ATP_ or C_NAD+_/C_NADH_, and “0” indicates that no controller ratio appears in the flux expression; ## denotes the estimated K_m_ value 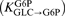 for the inhibition of hexokinase reaction by G6P.**

A detailed description of this robust estimation approach for obtaining optimal parameter estimates from species concentration dynamics during muscle ischemia and recovery is presented in Ref. [1]. This method has been established powerful in the analysis of large-scale *in vivo* metabolic systems. The efficiency and robustness of this parameter estimation approach was tested with parameter sensitivity analysis as well as with repeated parameter estimation with various initial parameter estimates (initial guesses). Various estimates of the more sensitive model parameters spanned in a small neighborhood of the optimal parameter estimates (see Ref. [1] for details). Since the preliminary estimates of the 12 transport parameters and 10 partition coefficients based on the resting species concentrations and transport fluxes were accurate enough, these 22 parameters were not re-estimated from the dynamic species concentration data. Therefore, as in our previous paper [1], a total of 101–22 = 79 parameters (77 reaction parameters+*V*_mus_+*Q*_isch_) are effectively estimated from the dynamic data.

## Results

### Comparison of model simulations with experimental data

The optimal estimates of the transport parameters (*λ*,*T*_max_,*M_m_*), partition coefficients (*σ*), active muscle volume and ischemic muscle blood flow (*V*_mus_, *Q*_isch_), and reaction parameters 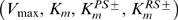 for the model are shown in Tables 3 and 4. These parameter estimates yield the best fit of the model outputs to the published experimental data [22] with minimal residual errors and minimal objective function. The optimal estimates of *V*_mus_ and *Q*_isch_ corresponding to the experimental data were ∼4.0 L (∼16% of normal two-legs muscle volume of ∼25 L) and ∼0.216 L/min (∼76% reduction from normal, resting two-legs muscle blood flow of *Q* = 0.9 L/min). These optimal parameter values were used for model simulations during the resting, ischemia and recovery periods. The blood flow reduction levels of *Q*_isch_ = 0.18, 0.27 and 0.36 L/min (80%, 70% and 60%) were used for simulating severe to moderate to mild ischemic conditions.

**Figure 4:**
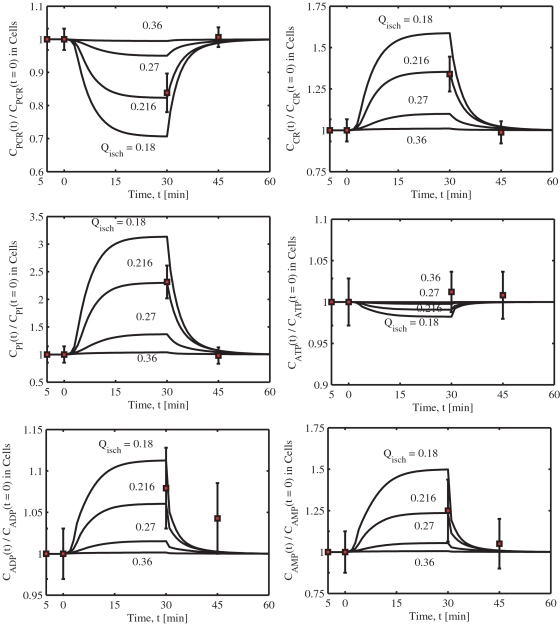
Model-predicted dynamic responses of energy metabolite concentrations in the muscle tissue cells during the resting, ischemia and recovery periods with varying levels of blood flow reductions and their comparison to the experimental data of Katz [22]. The lines represent the model simulation results with the symbols representing the experimental data points (mean±SD) corresponding to *Q*_isch_ = 0.216 L/min (∼76% blood flow reduction). The simulation strategy, ischemia protocol, and blood flow reduction levels are exactly the same as those described in the caption of Figure 3. The metabolites concentrations are shown in normalized form, normalized with respect to the resting metabolites concentrations. The concentrations in the tissue cells were calculated based on the formula: *C_cl_* = (*V_cyt_C_cyt_*+*V_mit_C_mit_*)/*V_cl_*.

**Figure 5:**
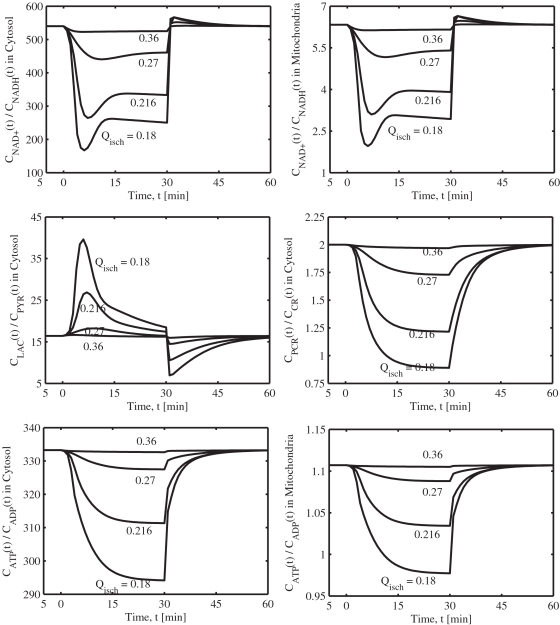
Model-predicted dynamic responses of cytosolic [LAC]/[PYR] and [PCR]/[CR] ratios and cytosolic and mitochondrial [ATP]/[ADP] and [NAD^+^]/[NADH] ratios during the resting, ischemia and recovery periods with varying levels of blood flow reductions. The simulation strategy, ischemia protocol, and blood flow reduction levels are exactly the same as those described in the caption of Figure 3.

The correspondence of model simulations and experimental data is demonstrated through Figures 3 and 4. Specifically, shown are the concentration dynamics of 4 glycolytic metabolites (GLC, G6P, LAC, PYR) and 6 energy metabolites (PCR, CR, PI, ATP, ADP, AMP) in the muscle tissue cells (i.e., weighted volume averages of concentrations in cytosol and mitochondria) for which experimental data were available [22] for four different blood flow reduction levels Q_isch_ = 0.18, 0.216, 0.27 and 0.36 L/min. Figure 3 also includes the concentration dynamics of NADH and NAD^+^ in the muscle tissue cells. The dynamics of compartmentalized cytosolic and mitochondrial phosphorylation and redox potentials (i.e., [ATP]/[ADP] and [NAD^+^]/[NADH] ratios) and cytosolic [LAC]/[PYR] and [PCR]/[CR] ratios are shown in Figure 5. The level *Q*_isch_ = 0.216 L/min corresponds to the experimental data of Katz [22] and the corresponding model simulations match to the data reasonably well within the experimental noise (Table 5). The experimental data are normalized here with respect to the resting metabolites concentrations (control). Furthermore, the individual metabolites responses are shown in separate plots in order to distinguish metabolite responses to different levels of blood flow reductions.

**Figure 6:**
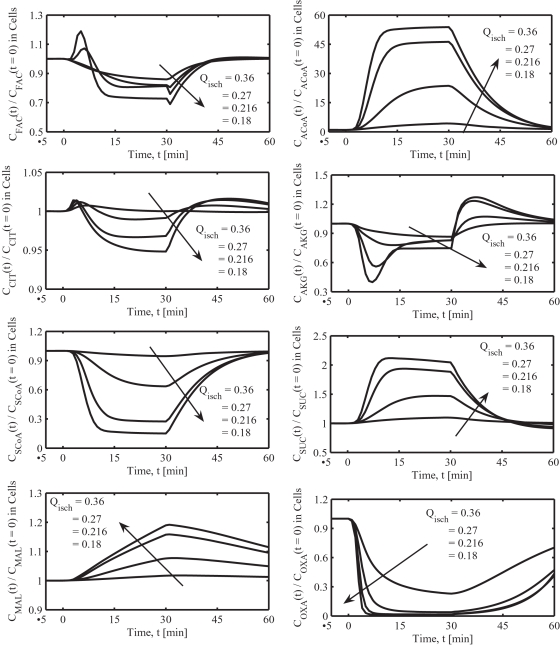
Model-predicted dynamic responses of FAC, ACoA, and TCA cycle intermediate concentrations in the muscle tissue cells mitochondria during the resting, ischemia and recovery periods with varying levels of blood flow reduction. The simulation strategy, ischemia protocol, and blood flow reduction levels are exactly the same as those described in the caption of Figure 3. The metabolites concentrations are shown in normalized form, normalized with respect to the resting metabolites concentrations. The concentrations in the tissue cells were calculated based on the formula: *C_cl_* = (*V_cyt_C_cyt_*+*V_mit_C_mit_*)/*V_cl_*.

**Table 5:** Model simulation results compared with the experimental data of Katz [22] on muscle ischemia.*

*Species or Species Ratio*	*Concentration Data (mM) (t = 0 min) (mean±SD)*	*Concentration Model (mM) (t = 0 min)*	*Concentration Data (mM) (t = 30 min) (mean±SD)*	*Concentration Model (mM) (t = 30 min)*	*Concentration Data (mM) (t = 45 min) (mean±SD)*	*Concentration Model (mM) (t = 45 min)*
GLC	0.485±0.0575	0.485	0.657±0.0925	0.676	0.653±0.0925	0.630
G6P	0.24±0.04	0.24	0.375±0.06	0.379	0.285±0.037	0.252
PYR	0.0425±0.006	0.0425	0.0925±0.0075	0.0918	0.0525±0.0075	0.057
LAC	0.688±0.12	0.688	1.535±0.38	1.545	0.94±0.12	0.850
LAC/PYR#	16.2±2.9	16.2	16.6±4.1	16.8	17.9±2.2	14.9
PCR	20.1±0.65	20.1	16.55±1.175	16.55	20.225±0.6	19.94
CR	10.45±0.7	10.45	14.0±1.1	14.12	10.325±0.7	10.62
PCR/CR#	1.97±0.12	1.97	1.35±0.13	1.17	2.03±0.15	1.88
Pi	2.70	2.7	6.25±0.8	6.2	2.6±0.3	2.845
ATP	6.15±0.175	6.15	6.225±0.15	6.092	6.2±0.175	6.148
ADP	0.82±0.025	0.82	0.885±0.04	0.869	0.855±0.035	0.822
AMP	0.04±0.005	0.04	0.05±0.007	0.0494	0.042±0.006	0.0403
TAN	7.0±0.175	7.01	7.05±0.125	7.01	7.1±0.175	7.01

* The model simulation are based on the ischemia protocol of Katz [22]: if (*t*<0 min | *t*>30 min) *Q* = 0.9 L/min, else *Q* = *Q*_isch_ = 0.216 L/min. The species concentrations (mM or mmol/kg ww) are converted from the experimental data (mmol/kg dw) by multiplying a factor 0.25 kg dw/kg ww [33]. For unit density, kg ww = L and mmol/kg ww = mmol/L = mM. The species concentrations are based on 100% muscle tissue cells, i.e., the volume averages of species concentrations in the cytosol and mitochondria, *C_cl_* = (*V_cyt_C_cyt_*+*V_mit_C_mit_*)/*V_cl_*. The species concentrations in the model are scaled with the resting (t = 0) species concentrations in the data (i.e., 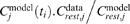) for comparison purpose; “dw” means dry weight and “ww” means wet weight.

# Concentration ratios [LAC]/[PYR] and [PCR]/[CR] are unitless.

The model simulations of cellular glucose (GLC), glucose-6-phosphate (G6P), pyruvate (PYR), and lactate (LAC) concentrations at the end of 76% ischemia and reperfusion are in close agreement with the experimental data (Fig. 3(A–D), Table 5). Cellular [G6P] increased quickly (exponentially) by ∼60% during ischemia and returned rapidly to its resting level at the onset of reperfusion. In contrast, the cellular [GLC] increased slowly (almost linearly) by ∼35% during ischemia and remained at an elevated level even after 30 minutes of reperfusion. Blood [GLC], however, decreased rapidly, but only by ∼10%, during ischemia and returned quickly to its baseline value during reperfusion (not shown). Furthermore, the model-predicted changes in the cellular [G6P] and [GLC] during the mild 60% and 70% blood flow reduction levels were not significant when compared to the changes during the high 76% and above (i.e., 80%) blood flow reduction levels.

Both cellular [LAC] and [PYR] increased by ∼125% during 76% ischemia and almost returned to the resting levels after 30 minutes of reperfusion. However, the dynamic response of [PYR] was different from that of [LAC]. During ischemia, [PYR] first decreased and then increased, but during reperfusion, [PYR] first sharply increased and then rapidly decreased to the resting level. On the other hand, [LAC] slowly (almost linearly) increased during ischemia and slowly (almost linearly) decreased during reperfusion. Blood [LAC] had the similar dynamics as that of cellular [LAC] (not shown). These differential dynamics of cellular [LAC] and [PYR] characterize the dynamics of cellular or cytosolic [LAC]/[PYR] ratio. The dynamics of cytosolic [LAC]/[PYR] ratio and cytosolic and mitochondrial redox states ([NADH] and [NAD^+^]) and redox potentials ([NAD^+^]/[NADH] ratios) were all similar having biphasic behaviors during the ischemia and reperfusion periods (Figs. 3(E,F) and 5(A–C)). This is in contrast to our previous model (in which the cytosolic and mitochondrial compartments were lumped into a single tissue cells compartment [1]) predictions that the dynamic responses of cellular [LAC]/[PYR] and [NAD^+^]/[NADH] ratios to ischemia and reperfusion are distinct. Thus the present compartmentalized model is able to correctly simulate the dynamic responses of cytosolic and cellular [LAC]/[PYR] ratios as well as cytosolic and mitochondrial [NAD^+^]/[NADH] ratios. The cytosolic [LAC]/[PYR] ratio rapidly increased from a resting level of ∼16.4 to ∼26.8 at the onset of 76% ischemia, then quickly decreased almost to the baseline value (∼17.22) as ischemia progressed, in agreement with the data (Table 5). In contrast, the cytosolic [NAD^+^]/[NADH] ratio decreased quickly from a resting level of ∼540 to ∼265 at the onset of 76% ischemia, then increased and reached a new steady state value of ∼332 as ischemia progressed. The LDH mass action ratio ([LAC][NAD^+^])/([PYR][NADH]) decreased considerably from a resting level of ∼8856 to ∼5585 during 76% ischemia. At different levels of blood flow reductions, the model-predicted changes were not proportional. The changes were negligible below 70% blood flow reduction levels and significant above 76% blood flow reduction levels.

The model simulations of muscle phosphocreatine (PCR), creatine (CR), and inorganic phosphate (PI) concentrations at the end of 76% ischemia and recovery are in good agreement with the experimental data (Fig. 4(A–C)). After 30 minutes, 76% ischemia resulted in ∼17.5% decrease in muscle PCR content, which was fully resynthesized after 30 minutes of recovery. Muscle CR and PI contents increased by ∼35% and ∼130%, respectively, at the end of 76% ischemia and returned to their resting levels at the end of recovery. The dynamics of [PCR] drop during ischemia and rise during recovery were exponential, but faster during the recovery period. The dynamics of both [CR] and [PI] show the opposite trends (both are mirror images of [PCR] having the similar time constants). The cytosolic [PCR]/[CR] ratio decreased substantially from a resting value of ∼2 to ∼1.2 during ischemia (Fig. 5D, Table 5). The model-predicted muscle [PCR], [CR], [PI] and [PCR]/[CR] ratio during the mild 60% and 70% blood flow reduction levels did not change appreciably from their baseline, resting levels.

The model-simulated muscle [ATP] decreased slightly during ischemia which resulted in appropriate increases in muscle [ADP] and [AMP] (Fig. 4(D–F)). A 76% blood flow reduction resulted in ∼5% and ∼25% increases in [ADP] and [AMP], respectively, in accordance with the data. The cytosolic and mitochondrial phosphorylation potentials ([ATP]/[ADP] ratios) had the similar dynamics as of the cytosolic [PCR]/[CR] ratio (Fig. 5(D–F)). However, the magnitude of [ATP]/[ADP] decrease (∼6%) during ischemia was negligible in comparison to the magnitude of [PCR]/[CR] decrease (∼40%). The CK mass action ratio ([CR][ATP])/([PCR][ADP]) increased from a resting value of ∼166 to ∼260 during the peak ischemia. The total adenylate nucleotide pool (TAN = [ATP]+[ADP]+[AMP]) was maintained at a constant level of ∼7.0 mM in accordance with the data (Table 5). The model-predicted changes in muscle [ATP], [ADP], [AMP], and [ATP]/[ADP] ratio during the mild 60% and 70% blood flow reduction conditions were negligible. Thus, in contrast to our previous model [1], the present compartmentalized model is able to simulate the dynamic responses of cytosolic and cellular [PCR]/[CR] ratios as well as cytosolic and mitochondrial [ATP]/[ADP] ratios appropriately.

### Simulated dynamics of mitochondrial metabolites

The model-simulated dynamic responses of mitochondrial FAC, ACoA, CIT, AKG, SCoA, SUC, MAL and OXA during the ischemia and recovery periods with blood flow reduction levels of 80%, 76%, 70% and 60% (Q_isch_ = 0.18, 0.216, 0.27 and 0.36 L/min) are shown in Figure 6. Experimental data were not available for these key mitochondrial and TCA cycle intermediate metabolites. In fact, due to the limitations in the available experimental techniques, most of these subcellular metabolites can not be measured conveniently in skeletal muscle tissue cells *in vivo* at the whole tissue-organ level. However, the present compartmentalized model is able to simulate the dynamic responses of these subcellular metabolites to physiological stresses such as muscle ischemia. Model simulations show that the changes in these metabolites concentrations during muscle ischemia are not simply proportional to the extent of blood flow reduction. The changes are negligible during the mild 60% and moderate 70% blood flow reduction levels, but significant above the severe 76% blood flow reduction level.

**Figure 7:**
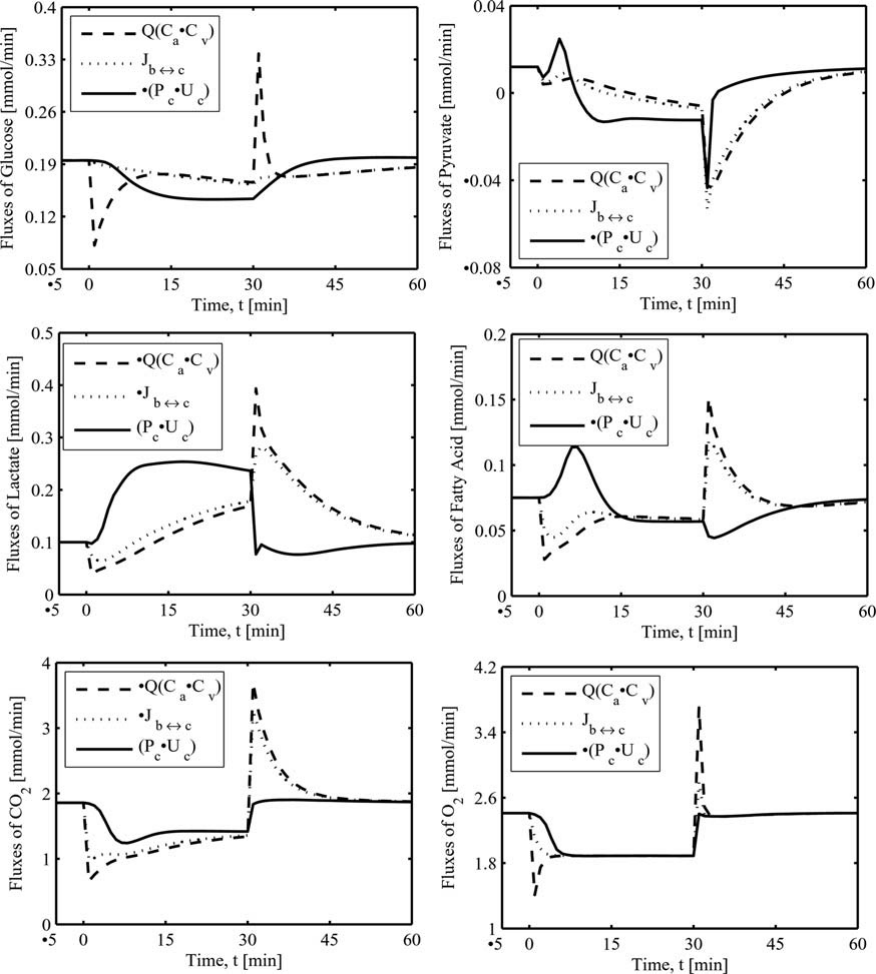
Model-predicted dynamic responses of transport and metabolic fluxes of glucose, pyruvate, lactate, free fatty acid, oxygen, and carbon dioxide during the resting, ischemia and recovery periods with a blood flow reduction of about 76%. The responses were computed using the estimated optimal parameter values with the ischemia protocol of −5 to 0 min of resting, 0 to 30 min of ischemia, and 30 to 60 min of recovery. The muscle blood flow Q is reduced as a step from 0.9 L/min at rest to Q_isch_ = 0.216 L/min at the onset of ischemia and returned to 0.9 L/min at the onset of recovery. Q(C_a_–C_v_): uptake-release rates, J_b↔c_: blood-tissue cells transport rates, P_c_–U_c_: tissue cells production-utilization rates.

The model-simulated dynamic responses of mitochondrial and TCA cycle intermediate metabolites to muscle ischemia differ from each other (Figure 6). During ischemia, the mitochondrial [ACoA], [SUC] and [MAL] were increased, while [CIT], [AKG] and [SCoA] were decreased. Furthermore, the accumulation of ACoA was very significant (∼45 fold increase in [ACoA] with 76% blood flow reduction), in spite of a large decrease in mitochondrial redox ratio [NAD^+^]/[NADH]. The accumulation of ACoA is primarily attributed to a biphasic increase in free [CoA] and a sharp 80-fold decrease in [OXA] (Fig. 6H) resulting in a mismatch in the reaction fluxes producing and consuming ACoA in the mitochondria. The decreases in [SCoA] and increases in [SUC] are in accordance with the increases in mitochondrial phosphorylation ratio [ADP]/[ATP]. However, the decreases in [CIT] and [AKG] and increases in [MAL] were not consistent with the increases in mitochondrial redox ratio [NAD^+^]/[NADH]. The TCA cycle reaction fluxes decreased from a resting level of ∼0.82 to ∼0.62 mmol/min (∼25%) with a 76% blood flow reduction (not shown).

### Simulated dynamics of fluxes of exchangeable substrates

**Figure 8 pone-0003168-g008:**
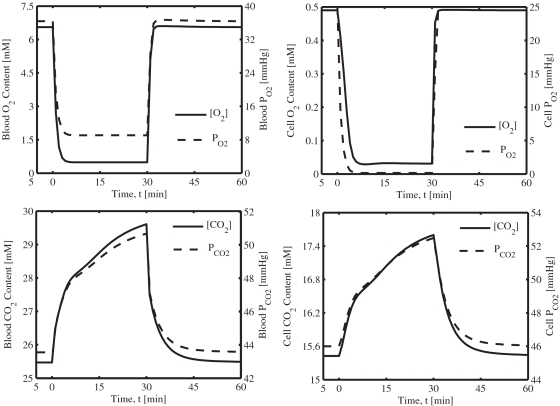
Model-predicted dynamic responses of O_2_ and CO_2_ (total contents and partial pressures) in the capillary blood (or venous blood) and tissue cells during the resting, ischemia and recovery periods with a blood flow reduction of about 76%. The responses were computed using the estimated optimal parameter values with the ischemia protocol: if (*t*<0 min | *t*>30 min) *Q* = 0.9 L/min, else *Q* = *Q*_isch_ = 0.216 L/min. The total O_2_ and CO_2_ contents are based on various forms of O_2_ and CO_2_ transports in the capillary blood and tissue cells (see Materials S1).

The model-simulated dynamic responses of transport and reaction rates associated with the blood-tissue cells exchangeable substrates (i.e., glucose, lactate, pyruvate, free fatty acid, CO_2_, and O_2_) during the resting steady-state (*t*<0 min), ischemia (0≤*t*≤30 min), and recovery (*t*>30 min) periods with a blood flow reduction level of 76% (*Q*_isch_ = 0.216 L/min) are shown in Figure 7. The corresponding capillary blood (venous blood) and tissue cells levels of O_2_ and CO_2_ are shown in Figure 8. The transport rates include the species net uptake/release rates (*UR_j_* = *Q*(*C_art,j_*−*C_ven,j_*)) and net blood-tissue cells exchange rates 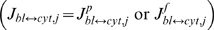. The reaction rates include the species net production/utilization rates (*R_cl,j_* = *P_cl,j_*−*U_cl,j_*) in the tissue cells (cytosol and/or mitochondria). Most of these model predictions are similar to those from our previous model [1] which do not account for the intracellular compartmentalization. The magnitudes of changes with the present compartmentalized model, however, are lower and consistent with the lower estimate of the blood flow reduction level during muscle ischemia (present *Q*_isch_ = 0.216 vs. previous *Q*_isch_ = 0.135).

**Figure 9:**
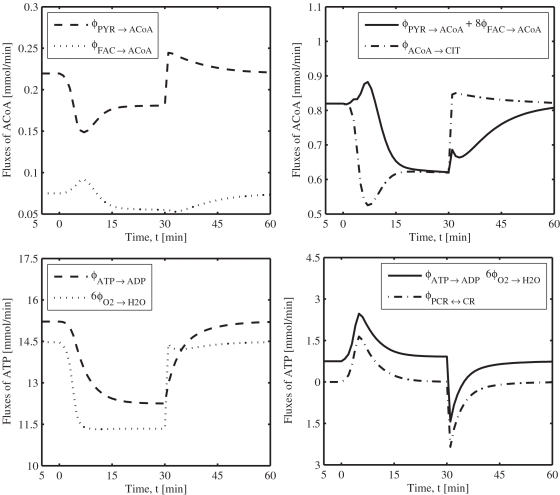
Model-predicted dynamic responses of metabolic reaction fluxes linked to the production and utilization of ACoA (A,B) and ATP (C,D) during the resting, ischemia and recovery periods with a blood flow reduction of about 76%. The responses were computed using the estimated optimal parameter values with the ischemia protocol: if (*t*<0 min | *t*>30 min) *Q* = 0.9 L/min, else *Q* = *Q*_isch_ = 0.216 L/min. The factors 8 and 6 in plots B and D correspond to the stoichiometries of ACoA and ATP in the production reactions of ACoA and ATP (reactions 18 and 26), respectively.

Figure 7A shows that the net muscle glucose uptake (*UR_GLC_*) changes sharply (decreases/increases) at the onset of ischemia/recovery due to a step change (decrease/increase) in muscle blood flow, while the net glucose uptake by muscle tissue cells (*J_bl↔cyt,GLC_*) remains fairly constant during ischemia and recovery. This results in a fast adjustment (decrease/increase) in the venous glucose concentration and glucose A–V difference and a rapid recovery in muscle net glucose uptake. The net glucose utilization (*U_cl,GLC_*−*P_cl,GLC_*) through the hexokinase reaction decreases/increases quickly during ischemia/recovery due to the increase/decrease of [G6P] and G6P inhibition of hexokinase reaction. This is reflected in the predicted elevated cellular [GLC] during recovery (Fig. 3A). Several inhibition mechanisms of hexokinase reaction by G6P were tested. A general uncompetitive inhibition mechanism, which effectively modifies the *K_m_* as well as *V*_max_ of the reaction [23], [31], was found to be the most suitable for the model to fit to the data.

The net pyruvate *UR* and *J_bl↔cyt_* rates are closely matched where both decrease/increase during ischemia/recovery, except at the onset of recovery, where *UR_PYR_* and *J_bl↔cyt,PYR_* rates first sharply decrease and then exponentially increase to the baseline level (Fig. 7B, dashed and dotted lines). The pyruvate metabolism switches from a net utilization to a net production and a net uptake to a net release during ischemia and vice-versa during recovery (as depicted through the solid line in Fig. 7B). Similar to pyruvate, the net *UR* and *J_bl↔cyt_* rates of lactate were approximately matched, through the −*J_bl↔cyt,LAC_* rate was slightly higher than the −*UR_LAC_* rate during ischemia and vice-versa during recovery (Fig. 7C, dashed and dotted lines). However, the net lactate release (−*UR_LAC_* and −*J_bl↔cyt,LAC_*) first decreases when muscle blood flow is reduced and then increases steadily (linearly) slightly above the baseline value during the remaining ischemic period. During recovery, the opposite, but more pronounced, trend occurs. The net lactate production (*P_cl,LAC_*−*U_cl,LAC_*) increased quickly at the onset of ischemia and reached a new steady-state level within the first 10 minutes of ischemia. At the onset of recovery, the net lactate production decreased quickly to the baseline value (solid line, Fig. 7C).

Figure 7D shows that the net *UR* and *J_bl↔cyt_* rates of free fatty acid (FFA) decreased quickly at the onset of ischemia and then increased rapidly towards the baseline value during ischemia (biphasic behavior); the opposite trend occurred during the recovery period. The net utilization of FFA (*U_cl,FFA_*−*P_cl,FFA_*) had exactly the opposite behavior of *UR_FFA_* and *J_bl↔cyt,FFA_* rates. The dynamics of CO_2_ release and FFA uptake were similar (dashed and dotted lines, Fig. 7(D,E)). However, the CO_2_ production was closely correlated to the O_2_ consumption (solid lines, Fig. 7(E,F)). The muscle O_2_ uptake, cellular O_2_ uptake, and cellular O_2_ consumption were all reduced during the ischemia period and were closely matched (all reduced by ∼22% during 76% ischemia), except at the onset of ischemia and recovery, where the muscle O_2_ uptake decreased/increased sharply with the step change (decrease/increase) in muscle blood flow and returned to the new steady state levels as ischemia progressed (Fig. 7F). The O_2_ partial pressure (*P*_O2_) in the muscle tissue cells decreased from 25 mmHg to ∼0.5 mmHg (i.e., about the *K_m_* value for O_2_ consumption), while the corresponding CO_2_ partial pressure (*P*_CO2_) increased from 46 mmHg to ∼52 mmHg during the 76% ischemia period (Fig. 8(B,D)). Accordingly, the total O_2_ content in the capillary blood (venous blood) decreased from ∼6.6 mM (∼36 mmHg) to ∼0.4 mM (∼8 mmHg) and the total CO_2_ content in the blood increased from ∼25.5 mM (∼43.6 mmHg) to ∼29.6 mM (∼51 mmHg) (Fig. 8(A,C)). These model simulations indicate that the muscle tissue cells were hypoxic during the 76% ischemia period; the reduced O_2_ consumption was maintained by the reduced O_2_ supply during ischemia.

### Simulated dynamics of fluxes associated with ACoA and ATP

The model-simulated dynamic responses of metabolic fluxes associated with the production and utilization of ACoA and ATP during the resting steady-state (*t*<0 min), ischemia (0≤*t*≤30 min) and recovery (*t*>30 min) periods with a blood flow reduction level of 76% (*Q*_isch_ = 0.216 L/min) are shown in Figure 9. Specifically, Figures 9A and 9B show the relative contributions from carbohydrates (PYR) and fats (FAC) to the production of ACoA in mitochondria, and the dynamics of ACoA utilization to CIT production in the mitochondrial TCA cycle. Figures 9C and 9D depict the utilization rate of ATP through ATP hydrolysis in cytosol and production rate of ATP through oxidative phosphorylation (O_2_ consumption) in mitochondria and creatine kinase buffer reaction in cytosol.

The production of mitochondrial ACoA from PYR decreased rapidly at the onset of ischemia and then increased relatively slowly to reach a new steady-state level as ischemia progressed (biphasic behavior). During recovery, the trend was opposite (Fig. 9A, dashed line). The production of mitochondrial ACoA from FAC varied relatively little during the entire ischemic and recovery periods (Fig. 9B, dotted line). An increase in total production of ACoA from PYR and FAC occurred at the beginning of ischemia due to a transient increase in the mitochondrial fatty acid β-oxidation. However, as ischemia progressed, ACoA production decreased and reached a new steady-state level significantly below its baseline value (Fig. 9B, solid line). In contrast, the utilization of ACoA to produce CIT decreased at the onset of ischemia and then increased to match the total ACoA production from PYR and FAC (Fig. 9B, dashed-dotted line). A mismatch between the total production and utilization of ACoA at the onset of ischemia is reflected in the predicted mitochondrial accumulation of ACoA during ischemia (Fig. 6B).

The rate of ATP utilization in cytosol through ATP hydrolysis slowly decreased (exponentially) by about 20% during 76% ischemia and returned to its baseline level during the recovery period (Fig. 9C, dashed line). However, the rate of ATP synthesis in mitochondria through oxidative phosphorylation (O_2_ utilization) rapidly decreased (as a step) by about 22% during 76% ischemia (Fig. 9C, dotted line) due to a rapid reduction in O_2_ delivery to mitochondria by capillary blood flow (Fig. 8B), in spite of sufficient increases in the mitochondrial redox potential [NADH]/[NAD^+^] (Fig. 5B) and phosphorylation potential [ADP]/[ATP] (Fig. 5F) (the activators of the lumped oxidative phosphorylation reaction). This transient deficit in ATP (Fig. 9D solid line) was mostly matched by the ATP production from the creatine kinase ATP buffer reaction (Fig. 9D, dashed-dotted line). The ATP supply from glycolysis/glycogenolysis (ATP deficit – ATP supply from creatine kinase reaction) during ischemia and recovery was almost constant, indicating that glycolysis/glycogenolysis is not a major source of ATP supply during partial ischemia. The time to reach new steady states (response time) for the ATP hydrolysis and creatine kinase reactions were about 15 minutes, while that for the ATP synthesis (or O_2_ consumption) reaction was about 5 minutes. Thus, in contrast to our previous model [1], the present compartmentalized model is able to correctly simulate the dynamic responses of cytosolic and mitochondrial metabolic reaction fluxes that are responsible in the production and utilization of ACoA and ATP. Furthermore, the model is able to link substrate metabolism (carbohydrates vs. fat) to energy metabolism in skeletal muscle during physiological perturbations such as muscle ischemia.

## Discussion

### Modeling framework for analysis of sparse *in vivo* experimental data

Analysis of dynamic changes in cellular metabolism and energetics in skeletal muscle *in vivo* in response to reduced blood flow and oxygen supply to mitochondria (ischemia) is limited by available *in vivo* experimental data. Relatively few *in vivo* measurements can be obtained of metabolite concentrations and metabolic fluxes in subcellular compartments, such as mitochondria. As a complementary approach to experimental measurements and as a framework for quantitatively analyzing available *in vivo* data, a physiologically-based, whole-organ level model of skeletal muscle cellular metabolism and energetics is developed. This model emphasizes a multi-scale, top-down systems approach [1]–[8] for quantitative understanding of metabolic processes at the molecular, subcellular and cellular levels in relation to the tissue-organ responses. For this purpose, integration of information was required from cellular metabolic pathways and fluxes of substrate and energy metabolism in cytosol and mitochondria, cellular metabolic control mechanisms, catalytic enzyme kinetic mechanisms, subcellular compartmentation and metabolites volumes of distribution, inter-domain transport mechanisms, and tissue-specific skeletal muscle metabolic characteristics. Since the extent to which enzyme activities and kinetic (Michaelis-Menten) constants determined from *in vitro* experimental studies are applicable to *in vivo* conditions is uncertain, our multi-scale, top-down modeling approach provides a unified, systematic framework for modeling and analysis of *in vivo* complex metabolic systems. A major improvement of the present model over our previous model [1] is the incorporation of distinct intracellular cytosolic and mitochondrial compartments. The present model distinguishes the cytosolic and mitochondrial [ATP]/[ADP] and [NADH]/[NAD^+^] ratios (major modulators of several key metabolic reactions in cytosol and mitochondria) and incorporates metabolic reactions and transport processes of key chemical species, including glucose, lactate, pyruvate, free fatty acid, oxygen and carbon dioxide (Fig. 1).

The governing model equations are based on dynamic mass balances of chemical species in spatially-lumped, capillary blood-interstitial fluid domain and two distinct intracellular cytosolic and mitochondrial domains (Fig. 2). These include the dynamic mass balances of O_2_ and CO_2_ which are developed by accounting for their distinct transport and binding mechanisms (hemoglobin and myoglobin-mediated) in these three domains [25], [26]. However, since not much information is available regarding the blood flow heterogeneity and fiber type distributions in skeletal muscle, these metabolic characteristics were not considered in the present study. Instead, different fiber types were lumped together to an effective active muscle volume with an effective blood flow supplying oxygen and other nutrients to the lumped muscle volume, which is sufficient to investigate the dynamic metabolic responses to muscle ischemia (also see discussions in Section 4.4). Furthermore, since the phenomenon of muscle acidification/alkalization (i.e., proton handling) under physiological stresses, such as muscle ischemia, is highly complex [17], [21], this was not accounted for in the present model.

In this model, the compartmentalized lumped biochemical reactions along the cellular metabolic pathways are the stoichiometrically coupled sequential elementary reactions, which include the compartmentalized metabolic energy controller pairs ATP-ADP and NADH-NAD^+^ whose ratios (phosphorylation and redox potentials) modulate (or fine tune) the reaction fluxes in the subcellular domains [2], [23]. The lumped reactions for which the resting Gibbs free energy (Δ*G*) is large and negative in favor of product formation are considered irreversible [24]. The reversible reactions like lactate dehydrogenase (LDH), creatine kinase (CK), and adenylate kinase (AK) are decomposed into two separate irreversible reactions with distinct kinetics. This can be justifiable based on the fact that the *in vivo* metabolic systems are nonequilibrium open systems and the biochemical reactions usually operate far from true equilibrium under physiological stimuli (also see discussions in Section 4.3). Besides, the consideration of the law of microscopic reversibility (thermodynamics) requires a detailed knowledge of proton handling in cytosol and mitochondria [17], [21], which is not considered in this phenomenological modeling study. The metabolic reaction fluxes in the subcellular cytosolic and mitochondrial domains are expressed in terms of a general phenomenological Michaelis-Menten equation with the coupled controller factors involving the compartmentalized [ATP]/[ADP] and [NADH]/[NAD^+^] energy controller ratios. These flux expressions characterize the behavior of saturable enzyme kinetics and *in vivo* control mechanisms observed experimentally [23], [31]. In comparison to the previous models in the literature [3]–[5], [11]–[21], the present model is a self-consistent, physiologically-based, multi-scale computational model dealing with cellular metabolism and energetics in skeletal muscle *in vivo* at the whole-organ level.

### Optimal parameter estimation for the large-scale metabolic model

A major challenge of this work was to evaluate a large number of unknown model parameters (precisely a total of 101) with relatively sparse experimental data from *in vivo* studies on muscle ischemia [22]. To deal with this under-determined, ill-conditioned problem, we had to devise a special optimization strategy (see Ref. [1] for details). Briefly, preliminary estimates of the model parameters were obtained based on resting, steady-state flux balance analysis together with the information on resting, steady-state metabolites blood and tissue cells (cytosol and mitochondria) concentrations, metabolites uptake-release (UR) rates or arterio-venous (AV) differences, and metabolic reaction flux rates gathered from various literature sources on skeletal muscle cellular metabolism in normal human subjects (Tables 1 and 2). With these preliminary parameter estimates, the model could not simulate the dynamic cellular metabolic responses to muscle ischemia. However, these estimates provided a good starting point in an iterative process of optimization to obtain the optimal parameter estimates (Tables 3 and 4) that yield the best least-squares fit of the model to the data [22] (Figs. 3 and 4). A good starting point is essential for optimization of large-scale problems with gradient-types of optimization algorithms, such as the generalized reduced gradient algorithm (GRG2) [34], used in the present study. The first step of obtaining the preliminary parameter estimates provided accurate optimal estimates of the 12 transport parameters (4 *λ*, 4 *T*_max_ and 4 *M*_m_) and 10 partition coefficients (10 *σ*), resulting in a reduction of 22 kinetic parameters for estimation from the dynamic experimental data from muscle ischemia.

The key to success of our parameter estimation approach for obtaining the remaining 79 kinetic parameters (second step) was the incorporation of constraint information to the maximum extent in the GRG2 optimization algorithm [34]. These include (1) the bound constraints on kinetic parameters from experimental studies, (2) the nonlinear equality (physiological) constraints relating fluxes and concentrations at resting, steady-state, and (3) the nonlinear inequality (thermodynamic) constraints relating fluxes and Gibbs free energy for reversible reactions: *φ*_net_.Δ*G*≤0 [35]–[37]. Also, the bounded estimation of active muscle volume (*V*_mus_) and ischemic blood flow (*Q*_isch_) (not known from the experimental protocol of Katz [22]) provided additional degrees of freedom for fitting the model to the data. Use of such constraints reduced the number of unknown model parameters for simultaneous estimation (e.g., the 26 *V*_max_ parameters could be explicitly estimated from the flux-concentration relationships at resting, steady-state). Such constraints also reduced uncertainty and enhanced accuracy in the estimated parameter values.

Since the available experimental data [22] were relatively sparse in comparison to the number of unknown model parameters, the parameter estimates for this under-determined, ill-conditioned problem were expected to be non-unique. The efficiency and robustness of this parameter estimation approach was tested previously [1] in detailed with repeated parameter estimations with various initial parameter estimates (guesses) and with parameter sensitivity analysis. Various parameter estimates of the more sensitivity model parameters spanned in a small neighborhood of the accepted optimal parameter estimates. Re-estimation of the model parameters for the present extended compartmentalized model with the same parameter estimation approach and the quality of the model fitting to the experimental data (Figs 3 and 4) further testifies the robustness of the parameter estimation approach.

### Model predictions related to muscle ischemia

This physiologically-based, multi-scale computational model of skeletal muscle cellular metabolism and energetics can simulate dynamic metabolic changes in cellular and subcellular compartments and provide quantitative analysis of the mechanisms of metabolic regulation during physiological stresses (e.g., reduced blood flow and oxygen supply to mitochondria associated with muscle ischemia). Model simulations based on the estimated optimal parameter values correspond well to the experimental data on muscle ischemia of Katz [22] (Figs. 3 and 4). The optimal estimates of active muscle volume *V*_mus_≈4 L and ischemic muscle blood flow *Q*_isch_≈0.216 L/min (Table 3) indicate that only ∼16% of whole muscle volume (∼25 L) was actively participating in cellular metabolism during reduced oxygen delivery to mitochondria induced by a blood flow reduction of ∼76% from the resting level of 0.9 L/min. Though these estimates are slightly different from those estimated previously [1] (*V*_mus_≈5 L and *Q*_isch_≈0.135 L/min), when scaled to one leg with a resting blood flow of 0.45 L/min, the estimated V_mus_ value becomes ∼2 L which lies well within the experimental range of 1.5–3 L for one leg quadriceps femoris muscle [38]–[41]. The experimental data [22] used in this computational study were also based on measurements from similar muscle types. This indicates that the whole muscle is not uniformly perfused and that only a fraction of the muscle mass, in accord with metabolic demand, participates in cellular metabolic processes.

The model simulations of cellular [GLC], [G6P], [LAC], [PYR], and [LAC]/[PYR] with ∼76% ischemia corresponded well with the experimental data (Fig. 3). To simulate [GLC] and [G6P] dynamics, and to some extent [LAC] and [PYR] dynamics in response to muscle ischemia, the model had to account for the inhibition of hexokinase reaction by [G6P] and activation of glycogen phosphorylase reaction by [AMP]/[ATP]. [G6P] increased during ischemia due to the increased [AMP]/[ATP] ratio and increased glycogenolysis flux. The increased [G6P] decreases the flux of hexokinase and glucose utilization. As a result, the glucose taken up by the muscle tissue cells is accumulated during ischemia.

The model-simulated muscle [PCR], [CR], [PCR]/[CR], [PI], [ATP], [ADP], [ATP]/[ADP], and [AMP] with ∼76% ischemia were in close agreement with the experimental data (Fig. 4). One of the key aspects in achieving these successful model simulations and good fittings was to model each of the reversible reactions like creatine kinase (CK) and adenylate kinase (AK) as two separate irreversible reactions with distinct kinetics. The forward and reversible reaction fluxes were considered regulated by the phosphorylation potentials [ATP]/[ADP] or [ADP]/[ATP] in the cytosol (Materials S2). Without considering such control mechanisms, it was not possible to simulate the experimental data on these high-energy metabolites during muscle ischemia and reperfusion.

We tested fitting the model to the data using alternative flux expressions for the CK and AK (also LDH) reversible reactions satisfying the Haldane relationship and thermodynamic equilibrium conditions. However, we were unable to achieve good fittings for the high-energy metabolites. This may be due to the limitations in the present model, or may be the fact that the data do not support thermodynamic equilibrium of the CK reaction during ischemia/recovery. It is evident from the data of Katz [22] that the changes in muscle [PCR]/[CR] and [ATP]/[ADP] ratios during ischemia are not proportional. The muscle [PCR]/[CR] ratio decreased substantially from a resting level of ∼2.0 to ∼1.2 (∼40%) during ischemia (Fig. 5D), while the muscle [ATP]/[ADP] ratio decreased only little from a resting level of ∼7.5 to ∼7.0 (∼7%). However, the dynamics of cytosolic [ATP]/[ADP] ratio was not available from the experimental study, which determines the thermodynamic equilibrium for the CK reaction. With our assumption of a constant cytosolic to mitochondrial ratio of [ATP] and [ADP] (and other common chemical species) during ischemia/recovery, the model predicted a comparable 7% decrease in cytosolic [ATP]/[ADP] ratio from a resting level of ∼332 to ∼310 during the ischemia period (Fig. 5E). As a consequence, the CK mass action ratio ([CR][ATP])/([PCR][ADP]) increased from a resting value of ∼166 to ∼260 during ischemia, instead of being a constant (∼166) (the CK apparent equilibrium constant at pH = 7) [17].

This issue may be resolved by including the dynamics and buffering of cytosolic and mitochondrial pH [17], [21] and, to some extent, by distinguishing the dynamics of common metabolites within the subcellular cytosolic and mitochondrial compartments. The later will necessitate incorporation of transport mechanisms of common metabolites across the mitochondrial inner membrane, e.g., proton pumps, ATP/ADP translocase, PI/H^+^ cotransporter, and exchange of TCA cycle substrates/intermediates [18], [21]. Since ∼98% of ADP is in mitochondria and only ∼2% of ADP is in cytosol at normal, resting steady-state conditions, a small relative change in whole cell [ADP] may correspond to a huge relative change in cytosolic [ADP], and hence a huge relative change in cytosolic [ATP]/[ADP] ratio. This can lead to the displacement of thermodynamic equilibrium of ATP/ADP translocase without displacing the thermodynamic equilibrium of CK reaction. Note that the cytosolic [ATP]/[ADP] ratio needs to decrease proportionally to the cytosolic [PCR][H^+^]/[CR] ratio for maintaining the thermodynamic equilibrium of the CK reaction. This information will be the mechanistic basis for the extension of the current model which can be used to analyze the data on high-energy phosphate metabolites.

With the estimated optimal parameter values, the present model is able to correctly simulate the dynamic metabolic responses of compartmentalized cytosolic and mitochondrial phosphorylation and redox potentials (Fig. 5), key mitochondrial and TCA cycle metabolite concentrations (Fig. 6), and other key metabolite concentrations and metabolic fluxes (Figs. 7–9) in skeletal muscle during ischemia and reperfusion for which no experimental data are available. Some of these simulations may be questionable without further validation, such as the predictions of cytosolic and mitochondrial phosphorylation and redox potentials (Fig. 5). However, these predictions are consistent with the dynamic responses of cellular or cytosolic [PCR]/[CR] and [LAC]/[PYR] ratios. Some of these simulations could not be obtained accurately with our previous model [1].

Our compartmentalized model simulations show that the metabolic changes were negligible with respect to the extent of blood flow reduction up to ∼70%, beyond which a profound derangement in substrate and energy metabolism occurred due to reduced supply of oxygen to mitochondria. The metabolic changes during severe ischemia (∼76% and beyond) include (1) a switch from a net pyruvate uptake and utilization to a net pyruvate production and release (Figs. 3C and 7B); (2) an increase in the redox potential [NADH]/[NAD^+^] (Figs. 3(E,F) and 5(A,B)) accompanied by a net formation and accumulation of cytosolic LAC and PYR (Fig. 3(C,D)) and mitochondrial ACoA (Figs. 6B and 9(A,B)); (3) an increase in the glycolytic and creatine kinase contributions to ATP formation (Fig. 9(C,D)) accompanied by a net depletion of cytosolic PCR (ATP stores) (Fig. 4A). In general, these predictions are in agreement with experimental results from studies under similar conditions [42]–[44]. However, since our model does not differentiate between FADH_2_ and NADH, the less efficient fuel (i.e., fats) –from the oxygen consumption point of view– is considered to have the same oxygen cost as carbohydrates for ATP production.

### Limitations of the model

In this multi-scale, multi-compartmental, top-down integrated model of cellular metabolism and energetics in skeletal muscle, the substrate and cation transport mechanisms between cytosol and mitochondria are not incorporated. Instead, these two domains are assumed to be in fast equilibrium with each other with the common metabolites having the similar dynamics (i.e., *C_mit,j_* = *σ_j_.C_cyt,j_*, with the partition coefficients *σ_j_* constant). This is projected in the model-simulated dynamic responses of cytosolic and mitochondrial phosphorylation and redox potentials (i.e., [ATP]/[ADP] and [NADH]/[NAD^+^] ratios) (Fig. 5). This assumption may be reasonable as a first approximation, since not much information is available regarding the dynamic responses of common metabolites to physiological stimuli (e.g., muscle ischemia) in these two subcellular domains (cytosol and mitochondria). This assumption may also be reasonable as long as the common metabolite concentrations do not vary appreciably within these two subcellular domains during physiological stresses. However, this assumption may not be valid for the [ATP]/[ADP] and [NADH]/[NAD^+^] ratios during ischemia/recovery, which are major modulators of several key metabolic reactions in cytosol and mitochondria. Therefore, the model may not accurately predict the dynamics of metabolite concentrations and metabolic fluxes that are critical in the regulation of fuel (carbohydrate, fat, and lactate) metabolism and cellular respiration during physiological perturbations, such as ischemia, hypoxia, and exercise. Furthermore, the inclusion of FADH_2_ as a reducing equivalent (produced through oxidation of fatty acids) is essential when investigating the relative contribution of carbohydrate and fat oxidations in the synthesis of ATP in mitochondria.

Another limitation in the present model is that the model does not account for blood flow heterogeneity and fiber type distributions associated with skeletal muscle cellular metabolism. Specifically, the capillary blood domain is considered spatially-lumped and the advective-diffusive transport of chemical species in capillary blood is ignored. Such compartmental modeling may not account for situations where important gradients of chemical species exist (e.g., due to distinct metabolic characteristics associated with different fiber types). In skeletal muscle, where the blood flow is highly heterogeneous due to complex capillarization and fiber type distributions, more than 70% of arterial oxygen is extracted from the blood before it leaves the microcirculation [45]. High extraction results in large intracellular and intravascular gradients of oxygen and other nutrients. Therefore, the well-mixed assumption may not be valid and compartmental modeling may not account for the heterogeneity of oxygen transport and consumption in different fiber types. These issues can be addressed through spatially-distributed modeling of oxygen transport and consumption, coupled with other substrates and CO_2_ [19], [26].

Finally, in the present model most of the lumped biochemical reactions in the cellular metabolic pathways are considered irreversible in the direction of product formation. Although the flux in the reverse direction is almost one order of magnitude smaller than the flux in the forward direction under normal, resting steady-state conditions, their magnitudes may change during physiological perturbations such as ischemia, hypoxia or exercise. Therefore, it would be advantageous to consider all the biochemical reactions to be reversible and their fluxes to satisfy the thermodynamic equilibrium constraints [37], including the dynamics and buffering of pH [17], [21], which can affect the apparent equilibrium constants and standard Gibbs free energy of the reactions.

### Future model developments and potential applications

Including the substrate and cation transport mechanisms between cytosol and mitochondria and distinguishing common metabolites dynamics within these subcellular domains would be important in the analysis of dynamic cellular metabolic responses to high intensity exercise. The subcellular compartmentalization could account for distinct volumes of metabolites distribution, for example, metabolic channeling for glycolysis [6]–[8]. This is a way to recognize that the key glycolytic enzymes are bound together to form a multi-enzyme complex near the sarcolemma and sarcoplasmic reticulum. To investigate the effects of spatial locality due to blood flow heterogeneity and fiber type distributions on metabolic responses to muscle ischemia, hypoxia or exercise, one will require spatially-distributed models involving parallally-arranged capillaries representing different muscle fiber types. Furthermore, the use of alternative kinetic flux expressions based on a Michaelis-Menten formalism for reversible enzymatic reactions would satisfy the thermodynamic equilibrium conditions and would provide additional thermodynamic constraints for kinetic parameter estimation [37]. As in our previous paper [1], a formal dynamic parameter sensitivity analysis can be carried out to understand the relative importance of the model parameters on the measured outputs. This information can be used to improve confidence in the estimates of key model parameters by fixing the values of the least sensitive parameters (parameter space reduction approach).

With these enhancements and with additional metabolic pathways similar to those of the models of cellular metabolism and energetics in cardiac muscle [6]–[8], such as the inclusion of FADH_2_ as a reducing equivalent, the advanced model of cellular metabolism and energetics in skeletal muscle can be used to analyze physiological responses to exercise (increased energy demand). Specifically, the model can assist in providing insight into long-standing or paradoxical questions, such as the extent of control that mitochondrial oxygen concentration and redox state exert over the rate of lactate production in the cytosol during heavy intensity exercise. In general, a multi-scale computational model of cellular metabolism and energetics in tissue/organ systems within the body that incorporate sufficient mechanistic processes can be used to (1) analyze and interpret *in vivo* experimental data; (2) provide new hypotheses as well as suggest how to test a given hypothesis; (3) design critical experiments; (4) quantify and predict dynamic responses to physiological stimuli that can not be directly measured; (5) evaluate relative importance of metabolic pathways and fluxes and their regulatory mechanisms under both normal and pathological conditions; and (6) provide the mechanistic basis for simulating integrated effects of altering enzyme activities and substrate concentrations through pharmacological agents and/or dietary inputs.

## Supporting Information

Materials S1 (0.34 MB DOC)

Materials S2 (0.43 MB DOC)

## References

[1] Dash RK, Li Y, Kim J, Saidel GM, Cabrera ME (2008). Modeling cellular metabolism and energetics in skeletal muscle: large-scale parameter estimation and sensitivity analysis.. IEEE Trans Biomed Eng.

[2] Cabrera M, Saidel G, Kalhan S (1998). Modeling metabolic dynamics from cellular processes to organ and whole body responses.. Prog Biophys Molec Biol.

[3] Cabrera M, Saidel G, Kalhan S (1998). Role of O_2_ in regulation of lactate dynamics during hypoxia: mathematical model and analysis.. Ann Biomed Eng.

[4] Cabrera M, Saidel G, Kalhan S (1999). Lactate metabolism during exercise: analysis by an integrative systems model.. Am J Physiol.

[5] Dash RK, Dibella JA, Cabrera ME (2007). A Computational Model of Skeletal Muscle Metabolism Linking Cellular Adaptations Induced by Altered Loading States to Metabolic Responses during Exercise.. Biomed Eng Online.

[6] Zhou L, Salem J, Saidel G, Stanley W, Cabrera M (2005). Mechanistic model of cardiac energy metabolism predicts localization of glycolysis to cytosolic subdomain during ischemia.. Am J Physiol Heart Circ Physiol.

[7] Zhou L, Stanley W, Saidel G, Yu X, Cabrera M (2005). Regulation of lactate production at the onset of ischemia is independent of mitochondrial NADH/NAD+: insights from in silico studies.. J Physiol.

[8] Zhou L, Cabrera ME, Okere IC, Sharma N, Stanley WC (2006). Regulation of myocardial substrate metabolism during increased energy expenditure: insights from computational studies.. Am J Physiol Heart Circ Physiol.

[9] Ainscow E, Brand M (1999). Internal regulation of ATP turnover, glycolysis and oxidative phosphorylation in rat hepatocytes.. Eur J Biochem.

[10] Brand M (1996). Top down metabolic control analysis.. J Theor Biol.

[11] Lai N, Dash RK, Nasca MM, Saidel GM, Cabrera ME (2006). Relating pulmonary oxygen uptake to muscle oxygen consumption at exercise onset: in vivo and in silico studies.. Eur J Appl Physiol.

[12] Lai N, Camesasca M, Saidel GM, Dash RK, Cabrera ME (2007). Linking Pulmonary Oxygen Uptake, Muscle Oxygen Utilization and Cellular Metabolism during Exercise.. Ann Biomed Eng.

[13] Korzeniewski B, Zoladz J (2001). A model of oxidative phosphorylation in mammalian skeletal muscle.. Biophys Chem.

[14] Korzeniewski B, Liguzinski P (2004). Theoretical studies on the regulation of anaerobic glycolysis and its influence on oxidative phosphorylation in skeletal muscle.. Biophys Chem.

[15] Kushmerick M (1998). Energy balance in muscle activity: Simulations of ATPase coupled to oxidative phosphorylation and to creatine kinase.. Comp Biochem Physiol B Biochem Mol Biol.

[16] Lambeth M, Kushmerick M (2002). Computational model for glycogenolysis in skeletal muscle.. Ann Biomed Eng.

[17] Vinnakota K, Kemp ML, Kushmerick MJ (2006). Dynamics of muscle glycogenolysis modeled with pH time course computation and pH-dependent reaction equilibria and enzyme kinetics.. Biophys J.

[18] Beard DA (2005). A biophysical model of the mitochondrial respiratory system and oxidative phosphorylation.. PLoS Comput Biol.

[19] Beard DA (2006). Modeling of Oxygen Transport and Cellular Energetics Explains Observations on In Vivo Cardiac Energy Metabolism.. PLoS Comput Biol.

[20] Wu F, Jeneson JA, Beard DA (2006). Oxidative ATP Synthesis in Skeletal Muscle is Controlled by Substrate Feedback.. Am J Physiol Cell Physiol.

[21] Wu F, Yang F, Vinnakota KC, Beard DA (2007). Computer modeling of mitochondrial tricarboxylic acid cycle, oxidative phosphorylation, metabolite transport, and electrophysiology.. J Biol Chem.

[22] Katz A (1988). G-1,6-P2, glycolysis, and energy metabolism during circulatory occlusion in human skeletal muscle.. Am J Physiol Cell Physiol.

[23] Fell D (1996). Understanding the Control of Metabolism.

[24] Nelson D, Cox M (2000). Lehninger Principles of Biochemistry (third edition).

[25] Dash RK, Bassingthwaighte JB (2004). Blood HbO_2_ and HbCO_2_ dissociation curves at varied O_2_, CO_2_, pH, 2,3-DPG and temperature levels.. Ann Biomed Eng.

[26] Dash RK, Bassingthwaighte JB (2006). Simultaneous blood-tissue exchange of oxygen, carbon dioxide, bicarbonate, and hydrogen ion.. Ann Biomed Eng.

[27] Shepherd PR, Kahn BB (1999). Glucose transporters and insulin action–implications for insulin resistance and diabetes mellitus.. N Engl J Med.

[28] Bonen A (2001). The expression of lactate transporters (MCT1 and MCT4) in heart and muscle.. Eur J Appl Physiol.

[29] Juel C, Halestrap AP (1999). Lactate transport in skeletal muscle - role and regulation of the monocarboxylate transporter.. J Physiol.

[30] Koonen DP, Glatz JF, Bonen A, Luiken JJ (2005). Long-chain fatty acid uptake and FAT/CD36 translocation in heart and skeletal muscle.. Biochim Biophys Acta.

[31] Segel I (1993). Enzyme Kinetics: Behavior and Analysis of Rapid Equilibrium and Steady-State Enzyme Systems.

[32] Radhakrishnan K, Hindmarsh (1993). Description and Use of LSODE, the Livermore Solver for Ordinary Differential Equations.

[33] Putman CT, Jones NL, Hultman E, Hollidge-Horvat MG, Bonen A (1998). Effects of short-term submaximal training in humans on muscle metabolism in exercise.. Am J Physiol Endocrinol Metab.

[34] Lasdon L, Warren A, Jain A, Ratner M (1978). Design and testing of a generalized reduced gradient code for nonlinear programming.. ACM Trans Math Software.

[35] Beard DA, Liang S, Qian H (2002). Energy balance for analysis of complex metabolic networks.. Biophysical Journal.

[36] Beard DA, Qian H (2005). Thermodynamic-based computational profiling of cellular regulatory control in hepatocyte metabolism.. Am J Physiol Endocrinol Metab.

[37] Beard DA, Qian H (2007). Relationship between thermodynamic driving force and one-way fluxes in reversible processes.. PLoS ONE.

[38] Gibala MJ, Gonzalez-Alonso J, Saltin B (2002). Dissociation between muscle tricarboxylic acid cycle pool size and aerobic energy provision during prolonged exercise in humans.. J Physiol.

[39] Gibala M, MacLean D, Graham T, Saltin B (1998). Tricarboxylic acid cycle intermediate pool size and estimated cycle flux in human muscle during exercise.. Am J Physiol.

[40] Radegran G, Blomstrand E, Saltin B (1999). Peak muscle perfusion and oxygen uptake in humans: importance of precise estimates of muscle mass.. J Appl Physiol.

[41] Ray C, Dudley G (1998). Muscle use during dynamic knee extension: implication for perfusion and metabolism.. J Appl Physiol.

[42] Chasiotis D, Hultman E (1983). The effect of circulatory occlusion on the glycogen phosphorylase-synthetase system in human skeletal muscle.. J Physiol.

[43] Timmons JA, Poucher SM, Constantin-Teodosiu D, Worrall V, MacDonald IA (1996). Metabolic responses of canine gracilis muscle during contraction with partial ischemia.. Am J Physiol Endocrinol Metab.

[44] Gutierrez G, Pohil RJ, Andry JM, Strong R, Narayana P (1988). Bioenergetics of rabbit skeletal muscle during hypoxemia and ischemia.. J Appl Physiol.

[45] Beard DA, Wu F, Cabrera ME, Dash RK (2008). Modeling of Cellular Metabolism and Microcirculatory Transport.. Microcirculation.

[46] Green HJ, Jones S, Ball-Burnett M, Farrance B, Ranney D (1995). Adaptations in muscle metabolism to prolonged voluntary exercise and training.. J Appl Physiol.

[47] Parolin M, Spriet L, Hultman E, Hollidge-Horvat M, Jones N (2000). Regulation of glycogen phosphorylase and PDH during exercise in human skeletal muscle during hypoxia.. Am J Physiol Endocrinol Metab.

[48] Phillips SM, Green HJ, Tarnopolsky MA, Heigenhauser GJ, Grant SM (1996). Progressive effect of endurance training on metabolic adaptations in working skeletal muscle.. Am J Physiol Endocrinol Metab.

[49] Turcotte LP, Richter EA, Kiens B (1992). Increased plasma FFA uptake and oxidation during prolonged exercise in trained vs. untrained humans.. Am J Physiol Endocrinol Metab.

[50] Sahlin K, Katz A, Henriksson J (1987). Redox state and lactate accumulation in human skeletal muscle during dynamic exercise.. Biochem J.

[51] Hurley BF, Nemeth PM, Martin WH, Hagberg JM, Dalsky GP (1986). Muscle triglyceride utilization during exercise: effect of training.. J Appl Physiol.

[52] Kiens B, Essen-Gustavsson B, Christensen NJ, Saltin B (1993). Skeletal muscle substrate utilization during submaximal exercise in man: effect of endurance training.. J Physiol.

[53] Sahlin K, Katz A, Broberg S (1990). Tricarboxylic acid cycle intermediates in human muscle during prolonged exercise.. Am J Physiol.

[54] Blomstrand E, Saltin B (1999). Effect of muscle glycogen on glucose, lactate, and amino acid metabolism during exercise and recovery in human subjects.. J Physiol.

[55] Howarth KR, Leblanc PJ, Heigenhauser GJ, Gibala MJ (2004). Effect of endurance training on muscle TCA cycle metabolism during exercise in humans.. J Appl Physiol.

[56] Van HG, Sacchetti M, Radegran G, Saltin B (2002). Human skeletal muscle fatty acid and glycerol metabolism during rest, exercise and recovery.. J Physiol.

[57] Wahren J, Felig P, Ahlborg G, Jorfeldt L (1971). Glucose metabolism during leg exercise in man.. J Clin Invest.

[58] Sahlin K, Poortmans JR (2004). High-Energy Phosphates and Muscle Energetics.. Principles of Exercise Biochemistry (Third edition).

[59] Ahlborg G, Felig P, Hagenfeldt L, Hendler R, Wahren J (1974). Substrate turnover during prolonged exercise in man.. J Clin Invest.

