# Supplementary material for: Metabolic Dynamics in Skeletal Muscle during Acute Reduction in Blood Flow and Oxygen Supply to Mitochondria: In-Silico Studies Using a Multi-Scale, Top-Down Integrated Model
Source: PLoS One. 2008 Sep 9;3(9):e3168. doi: 10.1371/journal.pone.0003168 (PMC2526172; doi:10.1371/journal.pone.0003168)
Supplement: Materials S1 — (0.34 MB DOC) [file pone.0003168.s001.doc]

# Materials S1: Dynamic Mass Balance Equations

**Capillary Blood + Tissue ISF Domain:** The dynamic mass balance equations for chemical species in the lumped capillary blood plus tissue ISF domain are identical to those of our previous model [1], except that the species blood-tissue cells exchange fluxes (*J_bl↔cyt,j_*) in the present model depend on the compartmentalized species cytosolic concentrations.

1.

2.

3.

4.

5.

6.

7.

8.

where the effective volumes (volumes of distributions) of O_2_ and CO_2_ in the capillary blood and tissue ISF ($V_{bl,\text{O2}}$, $V_{isf,\text{O2}}$, $V_{bl,\text{CO2}}$, $V_{isf,\text{CO2}}$) and total O_2_ and CO_2_ concentrations (contents) in the arterial and capillary blood ($C_{art,\text{CO2}}^{\text{T}}$, $C_{bl,\text{CO2}}^{\text{T}}$) can be derived by considering different forms of O_2_ and CO_2_ transport in blood and ISF [2–4] which are given by

$$V_{bl,O2}=V_{bl}\left(1+\frac{4.n_{H}.H_{rbc}.C_{rbc,Hb}.K_{HbO2}.\left(C_{bl,O2}^{F}\right)^{n_{H}-1}}{\left[1+K_{HbO2}.\left(C_{bl,O2}^{F}\right)^{n_{H}}\right]^{2}}\right), V_{isf,O2}=V_{isf}$$

$$C_{x,O2}^{T}=C_{x,O2}^{F}+\frac{4.H_{rbc}.C_{rbc,Hb}.K_{HbO2}.\left(C_{x,O2}^{F}\right)^{n_{H}}}{1+K_{HbO2}.\left(C_{x,O2}^{F}\right)^{n_{H}}}, x=(art,bl)$$

$V_{bl,CO2}=V_{bl}\left(1+\frac{4.H_{rbc}.C_{rbc,Hb}.K_{HbCO2}}{\left[1+K_{HbCO2}.C_{bl,CO2}^{F}\right]^{2}}+\left[\frac{(1-H_{rbc})}{C_{pl,H+}}+\frac{H_{rbc}}{C_{rbc,H+}}\right]K_{CO2hyd}\right)$, $V_{isf,CO2}=V_{isf}\left(1+\frac{K_{CO2hyd}}{C_{isf,H^{+}}}\right)$

$$C_{x,CO2}^{T}=C_{x,CO2}^{F}+\frac{4.H_{rbc}.C_{rbc,Hb}.K_{HbCO2}.C_{x,CO2}^{F}}{1+K_{HbCO2}.C_{x,CO2}^{F}}+\left[\frac{(1-H_{rbc})}{C_{pl,H+}}+\frac{H_{rbc}}{C_{rbc,H+}}\right]K_{CO2hyd}.C_{x,CO2}^{F}, x=(art,bl)$$

Here, *C_rbc,Hb_* = 5.2 mM is the concentration of hemoglobin in RBC, *H_rbc_* = 0.45 is the fraction of RBC in blood (hematocrits), *n_H_* = 2.7 is the Hill coefficient for HbO_2_ saturation, *K_HbO2_* = 7800.7 mM^–2.7^ is the Hill constant for HbO_2_ saturation, *K_HbCO2_* = 0.1237 mM^–1^ is the Hill constant for HbCO_2_ saturation, *K_CO2hyd_* = 7.95E-04 mM is the equilibrium constant for CO_2_ hydration reaction [2,3]. The pH in plasma, RBC, ISF and cells are approximately 7.4, 7.24, 7.2 and 7.1, respectively [3], and *C_H+_* = 10^–pH+3^ mM.

###### Tissue Cells (Cytosol and/or Mitochondria) Domain: **The dynamic mass balance equations for chemical species in the subcellular compartments (cytosol and/or mitochondria) are written here from equations (2a – 2d) of the manuscript. These equations differ from those of our previous model** [1] **through the changes in the metabolites volumes of distributions and metabolic reaction flux expressions due to compartmentalization. In the present model, the metabolic reaction fluxes are expressed in terms of the compartmentalized (cytosolic and mitochondrial) metabolites concentrations (see Appendix B)**.

1.

2.

3.

4.

5.

6.

7.

8.

9.

10.

11.

12.

13.

14.

15.

16.

17.

18.

19.

20.

21.

22.

23.

24.

25.

26.

27.

28.

29.

30.

Reference List

1. Dash RK, Li Y, Kim J, Saidel GM, Cabrera ME (2008) Modeling cellular metabolism and energetics in skeletal muscle: large-scale parameter estimation and sensitivity analysis. IEEE Trans Biomed Eng 55: 1298-1318.

2. Dash RK, Bassingthwaighte JB (2004) Blood HbO_2_ and HbCO_2_ dissociation curves at varied O_2_, CO_2_, pH, 2,3-DPG and temperature levels. Ann Biomed Eng 32: 1676-1693.

3. Dash RK, Bassingthwaighte JB (2006) Simultaneous blood-tissue exchange of oxygen, carbon dioxide, bicarbonate, and hydrogen ion. Ann Biomed Eng 34: 1129-1148.

4. Geers C, Gros G (2000) Carbon dioxide transport and carbonic anhydrase in blood and muscle. Physiol Rev 80: 681-715.

5. Nelson D, Cox M (2000) *Lehninger Principles of Biochemistry (third edition)*. Worth Publishers, New York.

6. Stryer L (1996) *Biochemistry (fourth edition)*. W.H. Freeman and Company, New York.

7. Segel I (1993) *Enzyme Kinetics: Behavior and Analysis of Rapid Equilibrium and Steady-State Enzyme Systems*. Wiley-Interscience, New York.
