# Supplementary material for: Metabolic Dynamics in Skeletal Muscle during Acute Reduction in Blood Flow and Oxygen Supply to Mitochondria: In-Silico Studies Using a Multi-Scale, Top-Down Integrated Model
Source: PLoS One. 2008 Sep 9;3(9):e3168. doi: 10.1371/journal.pone.0003168 (PMC2526172; doi:10.1371/journal.pone.0003168)
Supplement: Materials S2 — (0.43 MB DOC) [file pone.0003168.s002.doc]

# Materials S2: Metabolic Reactions Flux Expressions

The flux expressions for the compartmentalized lumped metabolic reactions that convert substrates to products in the two subcellular compartments (cytosol and mitochondria) in coupled with the energy controller pairs ATP-ADP and NADH-NAD^+^ are written here from the generalized reaction flux expression (equation (5) of the manuscript) which is based on a phenomenological one-step Michaelis-Menten kinetics for enzymatic reactions. These expressions differ from those of our previous model [1] through their dependencies on the compartmentalized (cytosolic or mitochondrial) metabolites concentrations, including the compartmentalized energy controller ratios ATP/ADP and NADH/NAD^+^. As necessary, further descriptions are given below for the individual reactions and the reaction flux expressions.

| **Reactions in Cytosol** | | | | | | | | | | | | |
| --- | --- | --- | --- | --- | --- | --- | --- | --- | --- | --- | --- | --- |
| 1. Glucose Utilization | | | $\text{GLC}+\text{ATP}\to\text{G6P}+\text{ADP}$ | | | | | | | | | |
| $\varphi_{\text{GLC}\to\text{G6P}}=\left(\frac{V_{\text{GLC}\to\text{G6P}}}{1+\frac{C_{\text{cyt,G6P}}}{K_{\text{GLC}\to\text{G6P}}^{\text{G6P}}}}\right)\left(\frac{\frac{C_{\text{cyt,ATP}}}{C_{\text{cyt,ADP}}}}{K_{\text{GLC}\to\text{G6P}}^{\text{PS+}}+\frac{C_{\text{cyt,ATP}}}{C_{\text{cyt,ADP}}}}\right)\left(\frac{\frac{C_{\text{cyt,GLC}}}{K_{\text{GLC}\to\text{G6P}}}}{1+\frac{C_{\text{cyt,GLC}}}{K_{\text{GLC}\to\text{G6P}}}+\frac{C_{\text{cyt,G6P}}}{K_{\text{GLC}\to\text{G6P}}^{\text{G6P}}}}\right)$ This reaction is catalyzed by the enzyme *hexokinase* which is inhibited by G6P ([5], Ch. 15; [6], Ch. 19). The exact inhibition mechanism *in-vivo* is not well known. Therefore, a combination of noncompetitive and product inhibition mechanism ([7], Ch. 3) is considered, which effectively modifies the *V*_max_ as well as the *K*_m_ of the reaction. | | | | | | | | | | | | |
| 1. Glycogen Synthesis | | | $\text{G6P}+\text{ATP}\to\text{GLY}+\text{ADP}+\text{2 PI}$ | | | | | | | | | |
| $\varphi_{\text{G6P}\to\text{GLY}}=V_{\text{G6P}\to\text{GLY}}\left(\frac{\frac{C_{\text{cyt,ATP}}}{C_{\text{cyt,ADP}}}}{K_{\text{G6P}\to\text{GLY}}^{\text{PS+}}+\frac{C_{\text{cyt,ATP}}}{C_{\text{cyt,ADP}}}}\right)\left(\frac{\frac{C_{\text{cyt,G6P}}}{K_{\text{G6P}\to\text{GLY}}}}{1+\frac{C_{\text{cyt,G6P}}}{K_{\text{G6P}\to\text{GLY}}}}\right)$ This is a sum of 4 enzymatic reactions G6P ↔ G1P, G1P+UTP → UDP-GLC + 2 PI, UDP-GLC + GLY_n_ → UDP + GLY_n+1_ and UDP+ATP → UTP+ADP catalyzed by the enzymes *phosphoglucomutase*, *UDP-glucose pyrophosphorylase*, *glycogen synthase*, and *nucleoside diphosphokinase* ([5], Ch. 20; [6], Ch. 23). | | | | | | | | | | | | |
| 1. Glycogen Breakdown | | | $\text{GLY}+\text{PI}\to\text{G6P}$ | | | | | | | | | |
| $\varphi_{\text{GLY}\to\text{G6P}}=V_{\text{GLY}\to\text{G6P}}\left(\frac{\frac{C_{\text{cyt,AMP}}}{C_{\text{cyt,ATP}}}}{K_{\text{GLY}\to\text{G6P}}^{\text{PS}-}+\frac{C_{\text{cyt,AMP}}}{C_{\text{cyt,ATP}}}}\right)\left(\frac{\frac{C_{\text{cyt,GLY}}C_{\text{cyt,PI}}}{K_{\text{GLY}\to\text{G6P}}}}{1+\frac{C_{\text{cyt,GLY}}C_{\text{cyt,PI}}}{K_{\text{GLY}\to\text{G6P}}}}\right)$ This is a sum of 2 enzymatic reactions GLY + PI → G1P and G1P ↔ G6P catalyzed by the enzymes *glycogen phosphorylase* and *phosphoglucomutase* ([5], Ch. 20; [6], Ch. 23). The activity of *glycogen phosphorylase* is regulated by AMP and ATP; AMP acts as a positive effector (activator) and ATP acts a negative effector (inhibitor) by competing with AMP. So the reaction is controlled by C_AMP_/C_ATP_ ratio. | | | | | | | | | | | | |
| 1. Glucose 6-Phosphate Breakdown | | | | | | | | | | $\text{G6P}+\text{ATP}\to\text{2 GA3P}+\text{ADP}$ | | |
| $\varphi_{\text{G6P}\to\text{GA3P}}=V_{\text{G6P}\to\text{GA3P}}\left(\frac{\frac{C_{\text{cyt,ATP}}}{C_{\text{cyt,ADP}}}}{K_{\text{G6P}\to\text{GA3P}}^{\text{PS+}}+\frac{C_{\text{cyt,ATP}}}{C_{\text{cyt,ADP}}}}\right)\left(\frac{\frac{C_{\text{cyt,G6P}}}{K_{\text{G6P}\to\text{GA3P}}}}{1+\frac{C_{\text{cyt,G6P}}}{K_{\text{G6P}\to\text{GA3P}}}}\right)$ This is a sum of 4 enzymatic reactions G6P ↔ F6P, F6P + ATP → F16BP + ADP, F16BP ↔ DHAP + GA3P, and DHAP ↔ GA3P catalyzed by the enzymes *phosphoglucose isomerase*, *phosphofructokinase*, *aldolase*, and *triose phosphate isomerase* ([5], Ch. 15; [6], Ch. 19). | | | | | | | | | | | | |
| 1. Glyceraldehyde 3-Phosphate Breakdown | | | | | | | | | | | | $\text{GA3P}+\text{PI}+\text{NAD}^{+}\to\text{13BPG}+\text{NADH}$ |
| $\varphi_{\text{GA3P}\to13\text{BPG}}=V_{\text{GA3P}\to13\text{BPG}}\left(\frac{\frac{C_{\text{cyt,NAD}^{\text{+}}}}{C_{\text{cyt,NADH}}}}{K_{\text{GA3P}\to13\text{BPG}}^{\text{RS}-}+\frac{C_{\text{cyt,NAD}^{\text{+}}}}{C_{\text{cyt,NADH}}}}\right)\left(\frac{\frac{C_{\text{cyt,GA3P}}C_{\text{cyt,PI}}}{K_{\text{GA3P}\to13\text{BPG}}}}{1+\frac{C_{\text{cyt,GA3P}}C_{\text{cyt,PI}}}{K_{\text{GA3P}\to13\text{BPG}}}}\right)$ This reaction is catalyzed by the enzyme *glyceraldehyde 3-phosphate dehydrogenase* and is known to be reversible ([5], Ch. 15; [6], Ch. 19). Since |ΔG| of the reaction is non-zero, we consider it as irreversible. | | | | | | | | | | | | |
| 1. Pyruvate Production | | | $\text{13BPG}+\text{2 ADP}\to\text{PYR}+\text{2 ATP }$ | | | | | | | | | |
| $\varphi_{\text{13BPG}\to\text{PYR}}=V_{\text{13BPG}\to\text{PYR}}\left(\frac{\frac{C_{\text{cyt,ADP}}}{C_{\text{cyt,ATP}}}}{K_{13\text{BPG}\to\text{PYR}}^{\text{PS}-}+\frac{C_{\text{cyt,ADP}}}{C_{\text{cyt,ATP}}}}\right)\left(\frac{\frac{C_{\text{cyt,13BPG}}}{K_{\text{13BPG}\to\text{PYR}}}}{1+\frac{C_{\text{cyt,13BPG}}}{K_{\text{13BPG}\to\text{PYR}}}}\right)$ This is a sum of 4 enzymatic reactions 13BPG + ADP ↔ 3PG + ATP, 3PG ↔ 2PG, 2PG ↔ PEP, and PEP + ADP → PYR+ATP catalyzed by the enzymes *phosphoglycerate kinase, phosphoglycerate mutase, enolase, and pyruvate kinase* ([5], Ch. 15; [6], Ch. 19). | | | | | | | | | | | | |
| 1. Pyruvate Reduction | | | $\text{PYR}+\text{NADH}\to\text{LAC}+\text{NAD}^{+}$ | | | | | | | | | |
| $\varphi_{\text{PYR}\to\text{LAC}}=V_{\text{PYR}\to\text{LAC}}\left(\frac{\frac{C_{\text{cyt,NADH}}}{C_{\text{cyt,NAD}^{\text{+}}}}}{K_{\text{PYR}\to\text{LAC}}^{\text{RS+}}+\frac{C_{\text{cyt,NADH}}}{C_{\text{cyt,NAD}^{\text{+}}}}}\right)\left(\frac{\frac{C_{\text{cyt,PYR}}}{K_{\text{PYR}\to\text{LAC}}}}{1+\frac{C_{\text{cyt,PYR}}}{K_{\text{PYR}\to\text{LAC}}}}\right)$ This is an important reaction in skeletal muscle metabolism. When oxygen availability is limited (e.g., during muscle ischemia or intense muscle activities), NADH in cytosol can accumulate and reduce pyruvate to lactate with the help of the enzyme *lactate dehydrogenase* ([5], Ch. 15; [6], Ch. 19). | | | | | | | | | | | | |
| 1. Lactate Oxidation | | | $\text{LAC}+\text{NAD}^{+}\to\text{PYR}+\text{NADH}$ | | | | | | | | | |
| $\varphi_{\text{LAC}\to\text{PYR}}=V_{\text{LAC}\to\text{PYR}}\left(\frac{\frac{C_{\text{cyt,NAD}^{\text{+}}}}{C_{\text{cyt,NADH}}}}{K_{\text{LAC}\to\text{PYR}}^{\text{RS-}}+\frac{C_{\text{cyt,NAD}^{\text{+}}}}{C_{\text{cyt,NADH}}}}\right)\left(\frac{\frac{C_{\text{cyt,LAC}}}{K_{\text{LAC}\to\text{PYR}}}}{1+\frac{C_{\text{cyt,LAC}}}{K_{\text{LAC}\to\text{PYR}}}}\right)$ This is the reverse *lactate dehydrogenase* reaction. During aerobic metabolism, NAD^+^ in cytosol can increase and oxidize lactate to pyruvate. | | | | | | | | | | | | |
| 1. Alanine Production | | |  | | | | | | | | | |
|  | | | | | | | | | | | | |
| 1. Triglyceride Synthesis | | | $\text{GA3P}+\text{3 FAC}+\text{ NADH}\to\text{TGL}+\text{3 CoA}+\text{ PI}+\text{NAD}^{+}$ | | | | | | | | | |
| $\varphi_{\text{GA3P}\to\text{TGL}}=V_{\text{GA3P}\to\text{TGL}}\left(\frac{\frac{C_{\text{cyt,NADH}}}{C_{\text{cyt,NAD}^{\text{+}}}}}{K_{\text{GA3P}\to\text{TGL}}^{\text{RS+}}+\frac{C_{\text{cyt,NADH}}}{C_{\text{cyt,NAD}^{\text{+}}}}}\right)\left(\frac{\frac{C_{\text{cyt,GA3P}}C_{\text{cyt,FAC}}}{K_{\text{GA3P}\to\text{TGL}}}}{1+\frac{C_{\text{cyt,GA3P}}C_{\text{cyt,FAC}}}{K_{\text{GA3P}\to\text{TGL}}}}\right)$ This is a sum of several enzymatic reactions. It can be viewed as lumping of reactions GA3P ↔ DHAP, DHAP + NADH → G3P + NAD^+^, and G3P + 3 FAC → TGL + 3 CoA + PI. The major enzymes are *glycerol-3 phosphate dehydrogenase* and *acyltransferase* ([5], Ch. 21; [6], Ch. 24). The synthesis of triglycerides from glycerol is neglected here as the activity of the enzyme *glycerol kinase* is negligible in muscle. | | | | | | | | | | | | |
| 1. Lipolysis (Triglycerides Hydrolysis) | | | | | | | | | | |  | |
|  | | | | | | | | | | | | |
| 1. Fatty Acyl-CoA Formation | | | | | | | | | | $\text{FFA}+\text{CoA}+\text{ 2 ATP}\to\text{FAC}+\text{ 2 ADP}+\text{2 PI}$ | | |
| $\varphi_{\text{FFA}\to\text{FAC}}=V_{\text{FFA}\to\text{FAC}}\left(\frac{\frac{C_{\text{cyt,ATP}}}{C_{\text{cyt,ADP}}}}{K_{\text{FFA}\to\text{FAC}}^{\text{PS+}}+\frac{C_{\text{cyt,ATP}}}{C_{\text{cyt,ADP}}}}\right)\left(\frac{\frac{C_{\text{cyt,FFA}}C_{\text{cyt,CoA}}}{K_{\text{FFA}\to\text{FAC}}}}{1+\frac{C_{\text{cyt,FFA}}C_{\text{cyt,CoA}}}{K_{\text{FFA}\to\text{FAC}}}}\right)$ This reaction is also called fatty acid activation in which the enzyme is *fatty acid thiokinase*. The activated fatty acid is transported into the mitochondrial matrix through the carnitine shuttle which is subsequently oxidized to ACoA by several enzymatic reactions ([5], Ch.21; [6], Ch.24). | | | | | | | | | | | | |
| 1. ATP Hydrolysis | | $\text{ATP}\to\text{ADP}+\text{PI}$ | | | | | | | | | | |
| $\varphi_{\text{ATP}\to\text{ADP}}=\left(\frac{V_{\text{ATP}\to\text{ADP }}}{1+\frac{C_{\text{cyt,PI}}}{K_{\text{ATP}\to\text{ADP}}^{\text{PI}}}}\right)\left(\frac{\frac{C_{\text{cyt,ATP}}}{C_{\text{cyt,ADP}}}}{K_{\text{ATP}\to\text{ADP}}^{\text{PS+}}+\frac{C_{\text{cyt,ATP}}}{C_{\text{cyt,ADP}}}}\right)$ This reaction is catalyzed by the enzyme *ATPase* and is the primary source of energy supply for muscle contraction ([5], Ch.14; [6], Ch.17). This reaction is inhibited by ADP and PI. | | | | | | | | | | | | |
| 1. Phosphocreatine Breakdown | | | | | | | | $\text{PCR}+\text{ADP}\to\text{CR}+\text{ATP}$ | | | | |
| $\varphi_{\text{PCR}\to\text{CR}}=V_{\text{PCR}\to\text{CR}}\left(\frac{\frac{C_{\text{cyt,ADP}}}{C_{\text{cyt,ATP}}}}{K_{\text{PCR}\to\text{CR}}^{\text{PS-}}+\frac{C_{\text{cyt,ADP}}}{C_{\text{cyt,ATP}}}}\right)\left(\frac{\frac{C_{\text{cyt,PCR}}}{K_{\text{PCR}\to\text{CR}}}}{1+\frac{C_{\text{cyt,PCR}}}{K_{\text{PCR}\to\text{CR}}}}\right)$ This is an ATP buffer reaction catalyzed by the enzyme *creatine kinase*. It functions to maintain ATP homeostasis during muscle contraction ([5], Ch.14; [6], Ch.17). It is the primary source of immediate energy supply during the transitions from rest to exercise. | | | | | | | | | | | | |
| 1. Phosphocreatine Synthesis | | | | | | | |  | | | | |
| $\varphi_{\text{CR}\to P\text{CR}}=V_{\text{CR}\to\text{PCR}}\left(\frac{\frac{C_{\text{cyt,ATP}}}{C_{\text{cyt,ADP}}}}{K_{\text{CR}\to\text{PCR}}^{\text{PS+}}+\frac{C_{\text{cyt,ATP}}}{C_{\text{cyt,ADP}}}}\right)\left(\frac{\frac{C_{\text{cyt,CR}}}{K_{\text{CR}\to\text{PCR}}}}{1+\frac{C_{\text{cyt,CR}}}{K_{\text{CR}\to\text{PCR}}}}\right)$ This is the reverse *creatine kinase* reaction where creatine is phosphorylated to phosphocreatine. | | | | | | | | | | | | |
| 1. Adenylate Kinase – Forward | | | | | | | | | |  | | |
|  | | | | | | | | | | | | |
| 1. Adenylate Kinase – Reverse | | | | | | | | | $\text{ADP}+\text{ADP}\to\text{AMP}+\text{ATP}$ | | | |
| $\varphi_{\text{ADP}\to\text{AMP}}=V_{\text{ADP}\to\text{AMP}}\left(\frac{\frac{C_{\text{cyt,ADP}}}{C_{\text{cyt,ATP}}}}{K_{\text{ADP}\to\text{AMP}}^{\text{PS-}}+\frac{C_{\text{cyt,ADP}}}{C_{\text{cyt,ATP}}}}\right)\left(\frac{\frac{C_{\text{cyt,ADP}}}{K_{\text{ADP}\to\text{AMP}}}}{1+\frac{C_{\text{cyt,ADP}}}{K_{\text{ADP}\to\text{AMP}}}}\right)$ | | | | | | | | | | | | |
| **Reactions in Mitochondria** | | | | | | | | | | | | |
| 1. Pyruvate Oxidation | | | $\text{PYR}+\text{CoA}+\text{NAD}^{+}\to\text{ACoA}+\text{NADH}+\text{CO}_{2}$ | | | | | | | | | |
| $\varphi_{\text{PYR}\to\text{ACoA}}=V_{\text{PYR}\to\text{ACoA}}\left(\frac{\frac{C_{\text{mit,NAD}^{\text{+}}}}{C_{\text{mit,NADH}}}}{K_{\text{PYR}\to\text{ACoA}}^{\text{RS}-}+\frac{C_{\text{mit,NAD}^{\text{+}}}}{C_{\text{mit,NADH}}}}\right)\left(\frac{\frac{C_{\text{mit,PYR}}C_{\text{mit,CoA}}}{K_{\text{PYR}\to\text{ACoA}}}}{1+\frac{C_{\text{mit,PYR}}C_{\text{mit,CoA}}}{K_{\text{PYR}\to\text{ACoA}}}}\right)$ This is the first reaction inside the mitochondrial matrix in which ACoA is formed from the oxidative decarboxylation of pyruvate (carbohydrate oxidation) by the enzyme *pyruvate dehydrogenase* leading to the TCA cycle ([5], Ch.16; [6], Ch.20). | | | | | | | | | | | | |
| 1. Fatty Acyl-CoA Oxidation | | | | | | $\text{FAC}+\text{7 CoA}+14\text{ NAD}^{+}\to\text{8 ACoA}+14\text{ NADH}$ | | | | | | |
| $\varphi_{\text{FAC}\to\text{ACoA}}=V_{\text{FAC}\to\text{ACoA}}\left(\frac{\frac{C_{\text{mit,NAD}^{\text{+}}}}{C_{\text{mit,NADH}}}}{K_{\text{FAC}\to\text{ACoA}}^{\text{RS}-}+\frac{C_{\text{mit,NAD}^{\text{+}}}}{C_{\text{mit,NADH}}}}\right)\left(\frac{\frac{C_{\text{mit,FAC}}C_{\text{mit,CoA}}}{K_{\text{FAC}\to\text{ACoA}}}}{1+\frac{C_{\text{mit,FAC}}C_{\text{mit,CoA}}}{K_{\text{FAC}\to\text{ACoA}}}}\right)$ This reaction producing ACoA from the activated fatty acid (fat) inside the mitochondrial matrix is highly complex. It is the result of combining 7 cycles of reactions in which each cycle consists of 4 enzymatic reactions catalyzed by the enzymes *acyl-CoA dehydrogenase*, *enoyl-CoA hydratase*, *beta-hydroxyacyl-CoA dehydrogenase*, and *acyl-CoA acetyletransferase* (*thiolase*) ([5], Ch. 21; [6], Ch. 24). For simplicity, the reducing equivalents FAD and FADH_2_ are considered equivalent to NAD^+^ and NADH, as they consume equal amount of O_2_. | | | | | | | | | | | | |
| 1. Citrate Production | | | | $\text{ACoA}+\text{OXA}\to\text{CIT}+\text{CoA}$ | | | | | | | | |
| $\varphi_{\text{ACoA}\to\text{CIT}}=V_{\text{ACoA}\to\text{CIT}}\left(\frac{\frac{C_{\text{mit,ACoA}}C_{\text{mit,OXA}}}{K_{\text{ACoA}\to\text{CIT}}}}{1+\frac{C_{\text{mit,ACoA}}C_{\text{mit,OXA}}}{K_{\text{ACoA}\to\text{CIT}}}}\right)$ This is the first reaction of TCA cycle catalyzed by the enzyme *citrate synthase* ([5], Ch. 16; [6], Ch. 20)*.* | | | | | | | | | | | | |
| 1. Alpha-Ketoglutarate Production | | | | | | | | | | $\text{CIT}+\text{NAD}^{+}\to\text{AKG}+\text{NADH}+\text{CO}_{2}$ | | |
| $\varphi_{\text{CIT}\to\text{AKG}}=V_{\text{CIT}\to\text{AKG}}\left(\frac{\frac{C_{\text{mit,NAD}^{\text{+}}}}{C_{\text{mit,NADH}}}}{K_{\text{CIT}\to\text{AKG}}^{\text{RS}-}+\frac{C_{\text{mit,NAD}^{\text{+}}}}{C_{\text{mit,NADH}}}}\right)\left(\frac{\frac{C_{\text{mit,CIT}}}{K_{\text{CIT}\to\text{AKG}}}}{1+\frac{C_{\text{mit,CIT}}}{K_{\text{CIT}\to\text{AKG}}}}\right)$ This is a sum of two enzymatic reactions CIT ↔ ICIT and ICIT+NAD^+^ → AKG+CO_2_+NADH catalyzed by the enzymes *aconitase* and *isocitrate dehydrogenase*. | | | | | | | | | | | | |
| 1. Succinyl-CoA Production | | | | | | |  | | | | | |
|  | | | | | | | | | | | | |
| 1. Succinate Production | | | | $\text{SCoA}+\text{GDP}+\text{PI}\to\text{SUC}+\text{CoA}+\text{GTP}$ | | | | | | | | |
| $\varphi_{\text{SCoA}\to\text{SUC}}=V_{\text{SCoA}\to\text{SUC}}\left(\frac{\frac{C_{\text{mit,ADP}}}{C_{\text{mit,ATP}}}}{K_{\text{SCoA}\to\text{SUC}}^{\text{PS}-}+\frac{C_{\text{mit,ADP}}}{C_{\text{mit,ATP}}}}\right)\left(\frac{\frac{C_{\text{mit,SCoA}}C_{\text{mit,PI}}}{K_{\text{SCoA}\to\text{SUC}}}}{1+\frac{C_{\text{mit,SCoA}}C_{\text{mit,PI}}}{K_{\text{SCoA}\to\text{SUC}}}}\right)$ Because the reaction GTP+ADP ↔ GDP+ATP is in fast equilibrium, we assume the GTP/GDP ratio proportional to the ATP/ADP ratio ([5], Ch. 16; [6], Ch. 20). | | | | | | | | | | | | |
| 1. Malate Production | | | | $\text{SUC}+\text{ NAD}^{+}\to\text{MAL}+\text{ NADH}$ | | | | | | | | |
| $\varphi_{\text{SUC}\to\text{MAL}}=V_{\text{SUC}\to\text{MAL}}\left(\frac{\frac{C_{\text{mit,NAD}^{\text{+}}}}{C_{\text{mit,NADH}}}}{K_{\text{MAL}\to\text{OXA}}^{\text{RS}-}+\frac{C_{\text{mit,NAD}^{\text{+}}}}{C_{\text{mit,NADH}}}}\right)\left(\frac{\frac{C_{\text{mit,SUC}}}{K_{\text{SUC}\to\text{MAL}}}}{1+\frac{C_{\text{mit,SUC}}}{K_{\text{SUC}\to\text{MAL}}}}\right)$ This is a sum of two enzymatic reactions SUC + FAD → FUM + FADH_2_ & FUM ↔ MAL catalyzed by the enzymes *succinate dehydrogenase* and *fumarate* ([5], Ch.16; [6], Ch.20); FAD and FADH_2_ are considered equivalent to NAD^+^ and NADH, as they consume equal amount of O_2_. | | | | | | | | | | | | |
| 1. Oxaloacetate Production | | | | |  | | | | | | | |
|  | | | | | | | | | | | | |
| 1. Oxygen Utilization | $\text{O}_{2}+6\text{ ADP}+6\text{ PI}+2\text{ NADH}\to\text{2 H}_{2}\text{O}+6\text{ ATP}+2\text{ NAD}^{+}$ | | | | | | | | | | | |
| $\varphi_{\text{O2}\to\text{H2O}}=V_{\text{O2}\to\text{H2O}}\left(\frac{\frac{C_{\text{mit,ADP}}}{C_{\text{mit,ATP}}}}{K_{\text{O2}\to\text{H2O}}^{\text{PS}-}+\frac{C_{\text{mit,ADP}}}{C_{\text{mit,ATP}}}}\right)\left(\frac{\frac{C_{\text{mit,NADH}}}{C_{\text{mit,NAD}^{\text{+}}}}}{K_{\text{O2}\to\text{H2O}}^{\text{RS}+}+\frac{C_{\text{mit,NADH}}}{C_{\text{mit,NAD}^{\text{+}}}}}\right)\left(\frac{\frac{C_{\text{mit,O2}}^{\text{F}}C_{\text{mit,PI}}}{K_{\text{O2}\to\text{H2O}}}}{1+\frac{C_{\text{mit,O2}}^{\text{F}}C_{\text{mit,PI}}}{K_{\text{O2}\to\text{H2O}}}}\right)$ This is also the ATP synthesis reaction. This is a sum of several enzymatic reactions at complex I–V that constitute the electron transport chain and oxidative phosphorylation inside the mitochondrial matrix ([5], Ch.19; [6], Ch.21); FAD and FADH_2_ are considered equivalent to NAD^+^ and NADH as they consume equal amount of O_2_. Furthermore, 1 NADH is assumed to produce 3 ATP (i.e., P/O ratio = 3; a perfect coupling with negligible proton leak). | | | | | | | | | | | | |

Reference List

1. Dash RK, Li Y, Kim J, Saidel GM, Cabrera ME (2008) Modeling cellular metabolism and energetics in skeletal muscle: large-scale parameter estimation and sensitivity analysis. IEEE Trans Biomed Eng 55: 1298-1318.

2. Dash RK, Bassingthwaighte JB (2004) Blood HbO_2_ and HbCO_2_ dissociation curves at varied O_2_, CO_2_, pH, 2,3-DPG and temperature levels. Ann Biomed Eng 32: 1676-1693.

3. Dash RK, Bassingthwaighte JB (2006) Simultaneous blood-tissue exchange of oxygen, carbon dioxide, bicarbonate, and hydrogen ion. Ann Biomed Eng 34: 1129-1148.

4. Geers C, Gros G (2000) Carbon dioxide transport and carbonic anhydrase in blood and muscle. Physiol Rev 80: 681-715.

5. Nelson D, Cox M (2000) *Lehninger Principles of Biochemistry (third edition)*. Worth Publishers, New York.

6. Stryer L (1996) *Biochemistry (fourth edition)*. W.H. Freeman and Company, New York.

7. Segel I (1993) *Enzyme Kinetics: Behavior and Analysis of Rapid Equilibrium and Steady-State Enzyme Systems*. Wiley-Interscience, New York.
